# Health and economic benefits of achieving hepatitis C virus elimination in Pakistan: A modelling study and economic analysis

**DOI:** 10.1371/journal.pmed.1003818

**Published:** 2021-10-19

**Authors:** Aaron G. Lim, Nick Scott, Josephine G. Walker, Saeed Hamid, Margaret Hellard, Peter Vickerman

**Affiliations:** 1 Population Health Sciences, Bristol Medical School, University of Bristol, Bristol, United Kingdom; 2 Burnet Institute, Melbourne, Australia; 3 Aga Khan University, Karachi, Pakistan; Boston University School of Public Health, UNITED STATES

## Abstract

**Background:**

Modelling suggests that achieving the WHO incidence target for hepatitis C virus (HCV) elimination in Pakistan could cost US$3.87 billion over 2018 to 2030. However, the economic benefits from integrating services or improving productivity were not included.

**Methods and findings:**

We adapt a HCV transmission model for Pakistan to estimate the impact, costs, and cost-effectiveness of achieving HCV elimination (reducing annual HCV incidence by 80% by 2030) with stand-alone service delivery, or partially integrating one-third of initial HCV testing into existing healthcare services. We estimate the net economic benefits by comparing the required investment in screening, treatment, and healthcare management to the economic productivity gains from reduced HCV-attributable absenteeism, presenteeism, and premature deaths. We also calculate the incremental cost-effectiveness ratio (ICER) per disability-adjusted life year (DALY) averted for HCV elimination versus maintaining current levels of HCV treatment. This is compared to an opportunity cost-based willingness-to-pay threshold for Pakistan (US$148 to US$198/DALY).

Compared to existing levels of treatment, scaling up screening and treatment to achieve HCV elimination in Pakistan averts 5.57 (95% uncertainty interval (UI) 3.80 to 8.22) million DALYs and 333,000 (219,000 to 509,000) HCV-related deaths over 2018 to 2030. If HCV testing is partially integrated, this scale-up requires an investment of US$1.45 (1.32 to 1.60) billion but will result in US$1.30 (0.94 to 1.72) billion in improved economic productivity over 2018 to 2030. This elimination strategy is highly cost-effective (ICER = US$29 per DALY averted) by 2030, with it becoming cost-saving by 2031 and having a net economic benefit of US$9.10 (95% UI 6.54 to 11.99) billion by 2050. Limitations include uncertainty around what level of integration is possible within existing primary healthcare services as well as a lack of Pakistan-specific data on disease-related healthcare management costs or productivity losses due to HCV.

**Conclusions:**

Investment in HCV elimination can bring about substantial societal health and economic benefits for Pakistan.

## Abstract 2

Aaron G Lim and colleagues model the health and economic benefits of eliminating hepatitis C in Pakistan.

## Author summary

**Why was this study done?:**

1. The World Health Organization (WHO) has set targets for the elimination of hepatitis C virus (HCV) as a public health problem by 2030.
2. Pakistan is believed to have the highest HCV burden globally and so is a priority country for achieving HCV elimination.
3. We have previously estimated that a direct investment of US$3.87 billion is required for HCV elimination in Pakistan, but the cost-effectiveness, as well as the economic benefits from integrating services and improvements to productivity, are unknown. Understanding these wider societal implications is important for informing decision-making.

**What did the researchers do and find?:**

4. We modelled the costs and cost-effectiveness of achieving HCV elimination in Pakistan through scaled-up service delivery, either stand-alone or with one-third of HCV testing integrated into existing healthcare infrastructure. Both scenarios were compared to status quo levels of HCV treatment.
5. The model found that HCV elimination averts substantial morbidity (5.57 million disability-adjusted life years or DALYs) and mortality (333,000 HCV-related deaths) over 2018 to 2030.
6. The HCV elimination strategy with partial integration is predicted to be highly cost-effective by 2030 (US$29 per DALY averted) and cost-saving by 2031 with a net economic benefit of US$9.10 billion by 2050.

**What do these findings mean?:**

7. Investment in HCV elimination by Pakistan is worthwhile as it brings substantial societal health and economic benefits; however, there is a trade-off between needing to invest early and the return on investment occurring later on.
8. Integrating HCV testing services into existing healthcare can reduce overall costs, although further studies are needed to quantify what level of integration is possible.

## Introduction

Recent scientific advances in hepatitis C virus (HCV) treatment have made HCV elimination achievable [1–6], with cure rates above 90% [7] using direct-acting antiviral (DAA) medications. DAAs are easy to administer, allowing treatment to occur in community settings by nonspecialists, rather than being limited to hospitals. In addition, simple prevention strategies such as supplying clean needles and syringes within healthcare settings and to people who inject drugs (PWID) can prevent new infections [8,9]. Due to these advances, the World Health Organization (WHO) developed its Global Health Sector Strategy (GHSS) on Viral Hepatitis in 2016 [10], providing targets for eliminating HCV as a global public health problem, including reducing the annual incidence of new chronic infections by 80% and mortality by 65% by 2030 compared to 2015 levels.

The vast majority of the global burden of HCV infections and associated mortality occurs in low- and middle-income country (LMIC) settings [11]. If the WHO HCV elimination targets are to be achieved by 2030, it is critical that there is scale-up of HCV testing and treatment in LMIC. There are multiple factors impacting on why LMIC are not committing to scaling up their HCV response, including the impact of COVID-19 [12]. Finite resources mean that countries have to make difficult choices as to what is prioritised in health budgets, highlighting the importance of countries knowing their HCV burden and the potential health and economic benefits of achieving HCV elimination.

Following the considerable scale-up of the Egyptian Government’s HCV elimination programme over recent years [13], Pakistan may now have the highest HCV burden globally, with chronic (or viraemic) HCV prevalence estimated at 3.7% [14,15] in 2018, equating to 7.3 million infections or about 10% of the global burden. Without treatment scale-up, this will lead to considerable morbidity and mortality, with modelling suggesting that 13.4 million new HCV infections and 1.4 million HCV-related deaths may occur by 2030 [16]. This expanding burden is due to ongoing HCV transmission in Pakistan, which is thought to be primarily spread through use of nonsterile medical and injecting equipment [16–19]. However, other community, social, and healthcare risk factors may also be important [16,19].

In Pakistan, national and provincial hepatitis control programmes have provided treatment to HCV-infected patients since 2005, with treatment numbers increasing to 150,000 to 160,000 per year in 2015 and DAAs becoming available in the public sector from 2016 onwards [20]. Previous modelling has shown that maintaining these treatment numbers would be insufficient for eliminating HCV in Pakistan [14,16]. Indeed, the scale-up in HCV screening and treatment needed to achieve the WHO elimination targets for reducing incidence by 80% requires 660,000 treatments per year at an investment of at least US$3.9 billion over 2018 to 2030 [14]. This includes the direct costs of case-finding (US$3.9 billion) and treatment (US$1.1 billion), as well as savings of US$1.1 billion from reduced healthcare costs from averted liver disease [14]. These previous analyses did not evaluate the additional health and economic benefits of achieving elimination, including improvements in health-related quality of life (HRQoL) and workforce productivity arising from reduced absenteeism and presenteeism, and cost efficiencies related to integrating HCV services within existing health services.

This study used modelling to estimate the disability-adjusted life years (DALYs) averted and net economic benefits, including productivity gains and cost-efficiencies from integrating services, of scaling up HCV screening and treatment to achieve the WHO HCV elimination targets for incidence in Pakistan.

## Methods

### HCV transmission model for Pakistan

We utilise an existing dynamic transmission model of HCV for Pakistan [14], incorporating key demographic, HCV transmission, and disease progression aspects, as well as a detailed cascade of care from screening through to treatment (model schematic in Fig A in S1 Text). Briefly, the model includes population growth, age stratifications (0 to 19, 20 to 29, 30+ years old), sex, injecting drug use, and liver disease progression states including decompensated cirrhosis (DC) and hepatocellular carcinoma (HCC). The model stratifies individuals by whether they are diagnosed or not (HCV antibody (Ab) and HCV RNA testing results), are recently screened (first-time or repeat Ab and/or RNA testing), are on HCV treatment, and whether they are successfully treated, resulting in a sustained virological response (SVR; effective cure), or not. The model also tracks individuals that are lost to follow-up (LTFU).

### Baseline model calibration

The baseline model was parameterised and calibrated using Pakistan-specific data, including HCV prevalence data from the 2007–2008 national survey [18], bio-behavioural surveys among PWID, blood donor HCV testing data over 1994 to 2014, and HCV treatment uptake pre-2018, as previously described [14] (Tables A-D in S1 Text). We randomly sampled 4,000 sets of model parameter values and calibration data estimates from their respective uncertainty distributions. For each model parameter set, unknown model parameters were estimated by calibrating the model to the sampled estimates for the chronic HCV prevalence by age and for PWID, and trends in overall HCV prevalence over time. Only parameter sets that produced model estimates within 95% CI of the overall chronic HCV prevalence from the national survey [18] were accepted, yielding 1,151 final model fits. All results are reported as median and 95% uncertainty intervals (UIs) across these 1,151 model fits.

### Cost and health utility

Costs arising from screening and treatment, offset by healthcare management savings (compared with status quo), are designated as direct costs. Within the baseline model, the costs of HCV screening and treatment (Table 1), inclusive of test kit costs, drug costs, staff time, and overheads were derived from a patient-level costing analysis [14,21] of a HCV treatment programme implemented by Médecins Sans Frontières (MSF) together with the local nongovernmental organisation SINA in Machar Colony, Karachi, over 2016 to 2017 [22,23] The annual healthcare costs of managing HCV-related disease (other than curative HCV treatment) for each HCV disease progression stage were also derived in this costing analysis (Table 1) [14,21].

**Table 1:** Direct testing, treatment, and healthcare management costs used in the modelling analyses. This includes the unit costs of HCV screening and treatment and estimated annual costs of managing chronic HCV infection by disease progression stage. These cost estimates are used in both intervention scenarios (status quo and elimination) and for each of the economic perspectives (A, B, and C). All costs are in 2018 US dollars (US$).

Direct Costs		Without Integration	With Partial Integration	Source/Comments
Screening/Diagnostics Costs*				
Ab test (each)				Costing analysis [21] from HCV treatment intervention at Machar Colony MSF-SINA clinic in Karachi, Pakistan [22,23].
	Ab-negative	US$8.38	US$5.72	
	Ab-positive	US$14.92	US$10.08	
PCR test (each)				
	PCR-negative	US$24.21	US$14.21	
	PCR-positive	US$30.97	US$20.97	
**Treatment Costs**				
Drug regimen costs				
	SOF+DCV (12-week supply)	US$18.00		Pakistan Health Research Council [14]
Visit costs				MSF-SINA costing analysis [21]. Simplified treatment pathway based on algorithm implemented by hospitals in Pakistan [45]. Laboratory costs for the partial integration pathway is US$10 cheaper assuming that the RNA test kit cost is US$5 instead of US$15 as negotiated in the Egyptian HCV elimination programme [13].
	Pre-cirrhosis	US$74.30		
	Post-cirrhosis	US$129.35		
Laboratory costs				
	Pre-cirrhosis	US$31.83	US$21.83	
	Post-cirrhosis	US$38.88	US$28.88	
Total treatment cost**				Sum of drug, visit, and laboratory costs.
	Pre-cirrhosis	US$124.13	US$114.13	12 weeks
	Post-cirrhosis	US$204.23	US$194.23	24 weeks
**Total Costs of Diagnosis and Treatment**				
	Pre-cirrhosis	US$170.02	US$145.18	Costs from above for positive Ab and PCR test, plus 12 weeks of DAA treatment
	Post-cirrhosis	US$250.12	US$225.28	Costs from above for positive Ab and PCR test, plus 24 weeks of DAA treatment
**Annual healthcare costs for managing HCV-related disease**				
	Pre-cirrhosis	US$14.80		Estimated using data from Cambodia [6] and adjusted to the Pakistan context [21] by applying WHO-CHOICE health service delivery costs for Pakistan [53].
	Compensated cirrhosis	US$46.70		
	DC	US$277.60		
	HCC	US$339.20		

*Screening/diagnostics costs include staff time and overheads, as well as the costs of diagnostics test kits.

**Total treatment costs include visit and laboratory costs, as well as the drug regimen costs of DAA therapy (12 weeks for pre-cirrhotic patients and 24 weeks for post-cirrhotic patients).

Ab, antibody; DAA, direct-acting antiviral; DC, decompensated cirrhosis; DCV, daclatasvir; HCC, hepatocellular carcinoma; HCV, hepatitis C virus; SOF, sofosbuvir.

Recent simplifications in delivering HCV testing suggest that it can be partially integrated into existing primary healthcare services (PHCS) provision [23], although the extent to which it can be done is uncertain. For a partially integrated approach, we assumed a scenario in which one-third of initial HCV Ab screening staff and overhead costs were integrated into regular healthcare activities. We also assumed a reduction in PCR cartridge cost from US$15 to US$5 as achieved in Egypt [13] (Table 1).

Disability weights for HCV-related disease progression stages were derived from the Global Burden of Disease (GBD) 2017 study [24,25] (Table 2) and used to calculate DALYs averted by HCV treatment.

**Table 2:** Productivity parameters and disability weights used in the model.

	Value and Range	Source/Comments
**Productivity parameters**		
Paid employment rate		
General population	Male: 77.2% (Range: 54.0%–100.0%) Female: 20.9% (Range: 14.6%–27.2%)	Pakistan Employment Trends 2018 report [30]
PWID	Same as male general population 77.2% (Range: 54.0%–100.0%)	Assumption based on 2016–2017 IBBS Survey among PWID [31], where almost all PWID were male (99.4%) and 76.6% of PWID were in paid employment [30].
Cost per year of productive life lost		
	US$1,443.63	Per capita gross domestic product for Pakistan from the World Bank [54]
Lost productivity attributable to hepatitis C		
Absenteeism	1.85% (Range: 1.30%–2.41%)*	US study [26] found that people with HCV had 4.88% absenteeism versus 3.03% for people without HCV. Higher rates assumed for people with cirrhosis (below).
Presenteeism	3.19% (Range: 2.19%–4.07%)*	US study [26] found that people with HCV had 16.69% presenteeism versus 13.50% for people without HCV.
Additional productivity losses for people with cirrhosis		
Absenteeism	2.79 times (Range: 1.95–3.63)*	European study [27] found that among HCV-infected, people with cirrhosis had 7.8% absenteeism versus 2.8% for people without cirrhosis.
Presenteeism	1.54 times (Range: 1.08–2.00)*	European study [27] found that among HCV-infected, people with cirrhosis had 13.1% presenteeism versus 8.5% for people without cirrhosis.
Relative reduction in absenteeism following SVR		
Cirrhotic	44.0% (Range: 30.8–57.2%)*	European study [27] found that absenteeism significantly improved in cirrhotic patients post-SVR, but not if non-cirrhotic.
Non-cirrhotic	No change in absenteeism post-SVR	
Relative reduction in presenteeism following SVR		
Cirrhotic	11.0% (Range: 7.7%–14.3%)*	European study [27] found that presenteeism improved to a larger degree in non-cirrhotic patients post-SVR.
Non-cirrhotic	20.0% (Range: 14.0%–26.0%)*	
Percentage of HCV-related deaths by age bracket		
15–29	2.5%	WHO 2016 estimates for Pakistan [32]. These age brackets are used for estimating the corresponding productivity gains from averted HCV-related deaths (see Methods B in S1 Text for details).
30–49	19.5%	
50–59	22.0%	
60+	55.9%	
**Disability weights (from Global Burden of Disease Study 2017** [24]**)**		
Pre-cirrhosis	0.011 (95% CI 0.005, 0.021)	No specific DALY weight available, so DALY weight for mild abdominopelvic problem was assumed [24]
Compensated cirrhosis	0.133 (95% CI 0.088, 0.190)	No specific DALY weight available, so DALY weight for severe acute HCV was assumed [24]
DC	0.178 (95% CI 0.123, 0.250)	DALY weight for DC of the liver due to HCV [24]
HCC	0.540 (95% CI 0.377, 0.687)	DALY weight for terminal phase of liver cancer due to HCV [24]

*All productivity parameters have ±30% uncertainty associated with them because they were based on data from other countries, and there is uncertainty as to how this may affect these estimates.

DALY, disability-adjusted life year; DC, decompensated cirrhosis; HCC, hepatocellular carcinoma; HCV, hepatitis C virus; PWID, people who inject drugs; SVR, sustained virological response.

### Productivity costs due to HCV infection

We extended the baseline model to estimate the indirect costs of lost productivity due to absenteeism (HCV-related sick days), presenteeism (people being less productive due to illness), and premature deaths. Evidence from the US suggested that HCV-infected individuals exhibit greater absenteeism (1.85% more) and presenteeism (3.19% more) than uninfected individuals [26] (Table 2). Moreover, data from 5 European countries [27] suggested greater absenteeism (2.79 times) and presenteeism (1.54 times) for people with cirrhosis compared to those without (Table 2). Achieving SVR reduces this absenteeism by 44.0% for people with cirrhosis, while presenteeism is reduced by 11.0% and 20.0% for people with and without cirrhosis, respectively (Table 2). Although no equivalent evidence exists for Pakistan or other LMICs, we felt that these estimates should still have some relevance in Pakistan, and so we allowed the estimates to vary uniformly by ±30% to account for uncertainty (Table 2).

To include these indirect costs using the human capital approach [28,29] (Methods A and B in S1 Text), years of productive life lost attributable to absenteeism among people with HCV before and after cure were calculated by multiplying the rate of absenteeism by the number of person-years affected by absenteeism and by the paid employment rate, assumed to be 77.2% and 20.9% among male and female adults in Pakistan [30]. The years of productive life lost attributable to presenteeism were calculated in a similar way. PWID were assumed to have the same employment rate as the male general population based on a recent study [31]. Years of potential productive life lost due to premature deaths were calculated by dynamically tracking a hypothetical cohort of people who died from HCV from their age at death until their assumed retirement age (60 years) or until they died from other causes [32] (Table 2, also see Methods B in S1 Text). To estimate total indirect costs, the years of potential productive life lost were multiplied by the per capita gross domestic product in Pakistan (US$1,443.63 in 2018).

DALYs and all costs were discounted at 3.5% per annum (based on reserve bank near-term GDP growth projections [33]; 0% and 7% tested in sensitivity analyses), with costs presented in 2018 US dollars (US$) (Table 1).

### Model impact and cost-effectiveness analyses

We compared the health outcomes and costs for the following modelled elimination (EL) scenario identified in our previous study [14] against a status quo (SQ) counterfactual from 2018 onwards.

#### Status quo (SQ) scenario

Assuming a range of referral rates for treatment (35% to 70%), this scenario calibrates a screening rate to mimic the SQ levels of 150,000 to 160,000 DAA treatments given annually.

#### Elimination (EL) scenario

To decrease incidence by 80% by 2030, this scenario includes one-time screening of the entire population over the first 5 years (2018 to 2022 inclusive), with prioritised testing in adults aged 30+ and PWID, then retesting in the general population every 5 years and PWID every year, with reengagement of those LTFU every 5 years. This scenario assumes that 90% of diagnosed individuals are referred to treatment, leading to on average 660,000 (95% UI 595,000 to 735,000) individuals being HCV treated annually.

For each scenario, we estimated the total number of people living with chronic HCV (overall and by disease stage) and the total number of DALYs by 2030 and 2050. We also estimated the direct costs of screening, treatment, and healthcare management, alongside the indirect cost savings from gained productivity. The net sum of direct and indirect costs is the net economic benefit, which can be positive, indicating monetary gain, or negative, indicating monetary loss. We considered 3 economic perspectives:

**Perspective A** only considers direct costs (testing, treatment, and healthcare management);

**Perspective B** includes indirect productivity gains in addition to direct costs; and

**Perspective C** includes a reduction in direct costs due to testing being partially integrated into existing health services alongside including indirect productivity gains.

Costs for each economic perspective were calculated from 2018 until 2030 and 2050. The cost-effectiveness of achieving HCV elimination was estimated for each perspective by calculating the incremental cost-effectiveness ratio (ICER; cost per DALY averted) for the EL scenario compared to the SQ scenario. This was compared to an estimated empirical health opportunity cost-based willingness-to-pay (WTP) threshold of US$148 to US$198 per DALY averted in 2018 for Pakistan [34]. This study is reported as per the Consolidated Health Economic Evaluation Reporting Standards (CHEERS) guidelines (Checklist A in S1 Text).

### Sensitivity analyses

Univariate sensitivity analyses were undertaken to evaluate how specific changes affected the year by which we achieve a positive net economic benefit (i.e., year by which HCV elimination strategy becomes cost-saving), assuming perspective C. We considered different assumptions on DAA medication costs (double or half the price at baseline), discount rate (none or double the discount rate at baseline), the cost of a productive life year lost (±20% of Pakistan’s GDP, or using median income), employment rate among PWID (halved employment if PWID) or those with end-stage liver disease (no employment if DC or HCC), reduction in absenteeism following SVR for pre-cirrhotic patients (44.0% reduction as with cirrhotic patients post-SVR), no disutility prior to DC, no healthcare management costs pre-cirrhosis, if less or greater integration were achieved (one-sixth or two-thirds of initial Ab screening compared to one-third), and if HCV elimination were to be achieved sooner (by 2025). Further details are in Table I in S1 Text.

## Results

### Impact and cost of status quo HCV treatment scenario

The model projects that maintaining SQ treatment rates will result in 8.99 (95% UI 8.12 to 10.00) million people with chronic HCV infection by 2030 with HCV incidence remaining stable at approximately 2015 levels. Over 2018 to 2030, the SQ scenario will result in 1.15 (95% UI 0.81 to 1.68) million HCV-related deaths and 24.06 (95% UI 18.58 to 31.42) million DALYs due to HCV (Table 3). This is expected to incur direct costs of US$5.00 (95% UI 4.53 to 5.48) billion over 2018 to 2030 if testing is not integrated (perspectives A and B), reducing to US$4.50 (95% UI 4.04 to 4.97) billion if it is integrated (perspective C). It is estimated that the SQ scenario leads to US$7.11 (95% UI 5.45 to 9.03) billion in lost productivity over 2018 to 2030 (perspectives B and C).

**Table 3:** Model projections of the HCV-related morbidity and mortality due to the SQ and EL scenarios over 2018 to 2030. DALYs are discounted at a rate of 3.5% per annum. The values represent the median and 95% UIs across 1,151 model fits.

2030 Estimates		SQ	EL
**People living with hepatitis C in 2030 (millions)**	Total	8.99 (8.12–10.00)	1.21 (1.05–1.39)
	Averted	--	7.78 (7.03–8.66)
	% Reduction	--	86.5% (85.5%–87.4%)
**Cumulative hepatitis C–related deaths 2018–2030**	Total	1,153,000 (811,000–1,678,000)	821,000 (589,000–1,105,000)
	Averted	--	333,000 (219,000–509,000)
	% Reduction	--	28.9% (25.2%–33.1%)
Total DALYs* 2018–2030 (millions)	Total	24.06 (18.58–31.42)	18.53 (14.61–23.43)
	Averted	--	5.57 (3.80–8.22)
	% Reduction	--	23.2% (19.6%–27.5%)

*Total DALYs = Years Lived with Disability (YLD) + Years of Life Lost (YLL).

DALY, disability-adjusted life year; EL, elimination; HCV, hepatitis C virus; SQ, status quo; UI, uncertainty interval.

### Impact of HCV elimination scenario

**Fig 1:**
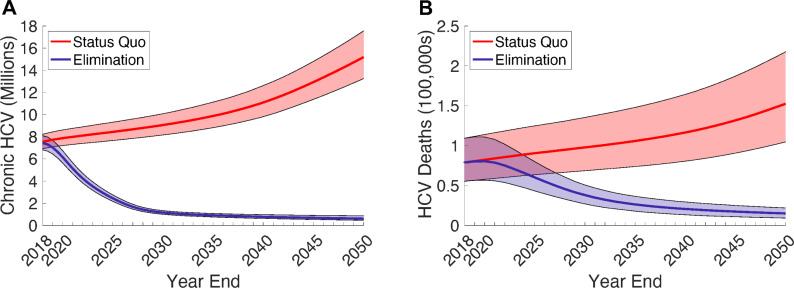
Estimated health impact of the SQ and EL scenarios on (A) the projected number of people living with hepatitis C and (B) the number of annual hepatitis C–related deaths. The solid line and shading indicate the median and 95% UIs across 1,151 model fits. EL, elimination; HCV, hepatitis C virus; SQ, status quo; UI, uncertainty interval.

Compared to the SQ scenario, scaling up screening and treatment to achieve an 80% reduction in incidence by 2030 (EL) is estimated to reduce the number of people with chronic HCV infection by 86.5% (95% UI 85.5% to 87.4%) to 1.21 (95% UI 1.05 to 1.39) million by 2030 (Fig 1A). This will avert 5.57 (95% UI 3.80 to 8.22) million DALYs and 333,000 (95% UI 219,000 to 509,000) HCV-related deaths (Fig 1B) between 2018 and 2030.

### Costs of HCV elimination scenario

**Fig 2:**
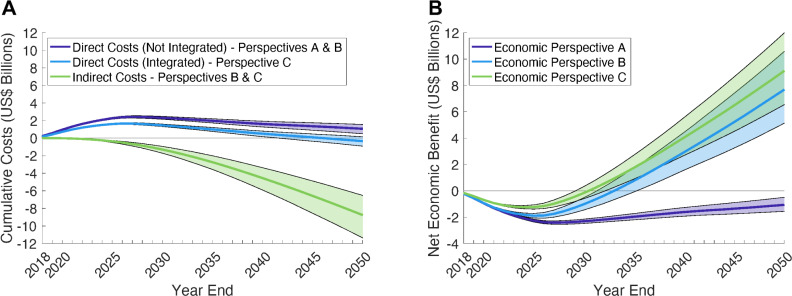
(A) Estimated cumulative direct costs and indirect costs of EL versus SQ. (B) Net economic benefit of EL versus SQ including direct and indirect costs. For the net economic benefit of EL, 3 economic perspectives were used: Perspective A–Direct costs only with no integration of testing. Perspective B–Direct costs (no integration of testing) and productivity gains. Perspective C–Partially integrated direct costs and productivity gains. All costs are in 2018 US$ and discounted at 3.5% per annum; healthcare costs were applied to all liver disease states pre- and post-cure; staffing costs were applied to all testing and treatment interactions; one-third of initial screening was assumed to not incur staffing costs and had reduced HCV RNA testing kit cost in the EL scenario with economic perspective C. The solid line and shading indicate the median and 95% UIs across 1,151 model fits. EL, elimination; HCV, hepatitis C virus; SQ, status quo; UI, uncertainty interval.

From economic perspective A (direct costs only), the EL scenario is estimated to cost an additional US$2.31 (95% UI 2.15 to 2.47) billion in direct costs for screening, treatment, and healthcare management, compared to the SQ over 2018 to 2030 (Fig 2A and Table 4) [14]. Additionally including gains in productivity costs (perspective B) reduces the total cost by US$1.30 (95% UI 0.94 to 1.72) billion to US$1.01 (95% UI 0.52 to 1.45) compared to the SQ (Table 4 and Table G in S1 Text). The gains in productivity costs continue to accumulate over time, with the EL scenario becoming cost-saving by 2033 (95% UI 2031 to 2035) and there being a net economic benefit of US$7.68 (95% UI 5.13 to 10.58) billion by 2050 (Fig 2B). Lastly, including the savings from partial integration of HCV Ab testing into existing services (perspective C) reduces the total direct costs to US$1.45 (95% UI 1.32 to 1.60) billion over 2018 to 2030 (Fig 2A), with this nearly being offset by the gains in productivity costs of US$1.30 billion. From this perspective C, the EL scenario becomes cost-saving by 2031 (95% UI 2029 to 2032), with a projected net economic benefit of US$9.10 (95% UI 6.54 to 11.99) billion by 2050 (Fig 2B).

**Table 4:** ICERs for the modelled EL scenario over 2018–2030 for 3 economic perspectives. Costs and DALYs are discounted at 3.5% per annum. Perspective A includes direct costs only (costs for testing, treatment, and healthcare management). Perspective B includes direct costs (perspective A) plus productivity costs. Perspective C includes partially integrated direct costs and productivity costs. The values represent the median and 95% UIs across 1,151 model fits.

	Costs (US$ billions)		DALYs (millions)		ICER	Probability	
Scenarios until 2030	Total	Incremental	Total	Incremental DALYs averted	Cost/DALY averted	Cost-effective^†^	Cost-saving
**Perspective A**							
**SQ**	US$5.00 (4.53 to 5.48)	--	24.06 (18.58 to 31.42)	--	--	--	--
**EL**	US$7.32 (6.80 to 7.78)	US$2.31 (2.15 to 2.47)	18.53 (14.61 to 23.43)	5.57 (3.80 to 8.22)	US$417	0%	0%
**Perspective B**							
**SQ**	US$12.09 (10.31 to 14.19)	--	24.06 (18.58 to 31.42)	--	--	--	--
**EL**	US$13.12 (11.69 to 14.85)	US$1.01 (0.52 to 1.45)	18.53 (14.61 to 23.43)	5.57 (3.80 to 8.22)	US$181	33.4% (Lower) 58.2% (Higher)	0%
**Perspective C**							
**SQ**	US$11.60 (9.82 to 13.68)	--	24.06 (18.58 to 31.42)	--	--	--	--
**EL**	US$11.77 (10.36 to 13.49)	US$0.16 (−0.33 to 0.59)	18.53 (14.61 to 23.43)	5.57 (3.80 to 8.22)	US$29	98.0% (Lower) 99.9% (Higher)	25.3%

^**†**^Compared to estimated empirical health opportunity cost-based WTP threshold of US$148–US$198 per DALY averted in 2018 for Pakistan [34]. The lower estimates are compared to the lower value of this range, while the higher estimate is compared to the higher value of this range.

DALY, disability-adjusted life year; EL, elimination; ICER, incremental cost-effectiveness ratio; SQ, status quo; UI, uncertainty interval; WTP, willingness-to-pay.

### Cost-effectiveness of HCV elimination scenario

Compared to SQ, the EL scenario is estimated to have an ICER of US$417 per DALY averted by 2030 (Table 4) when considering only direct costs (perspective A). The ICER reduces to US$181 and US$29 per DALY averted by 2030 when also including productivity gains (perspective B) and partial integration (perspective C), respectively (Table 4 and Fig D in S1 Text). With perspective C, the ICER has a 25.3% probability of being cost-saving and a 98.0% probability that it is below the health opportunity WTP threshold for Pakistan (US$148 to US$198 per DALY averted) (Table 4). Conversely, for perspectives A and B, there is a 0% and 33.4% chance that the ICER is below this threshold, respectively.

### Sensitivity analyses

**Fig 3:**
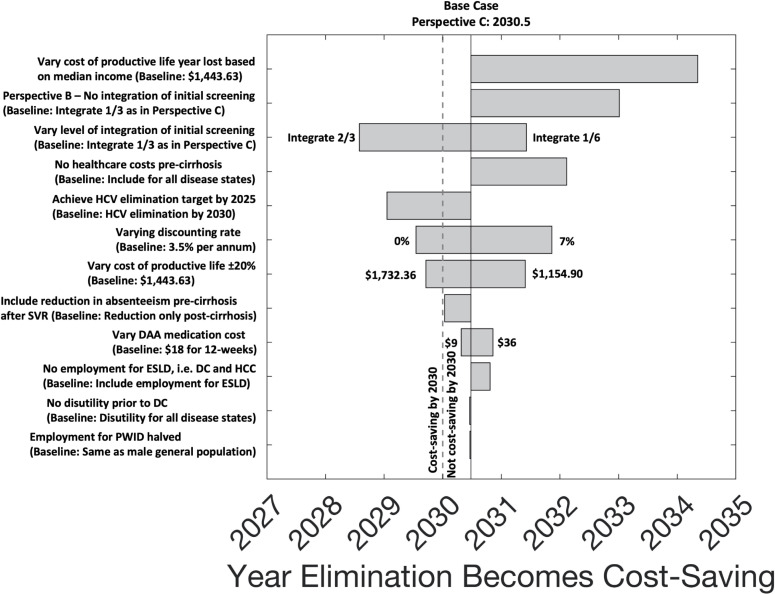
Univariate sensitivity analyses on the year that the HCV EL scenario becomes cost-saving. For each sensitivity analysis scenario, the estimated year that HCV elimination becomes cost-saving or, equivalently, the year when overall net economic benefit becomes positive, is taken from economic perspective C, compared to SQ. The bars show the median across 1,151 model runs for the various sensitivity analyses. DAA, direct-acting antiviral; DC, decompensated cirrhosis; EL, elimination; ESLD, end-stage liver disease; HCC, hepatocellular carcinoma; HCV, hepatitis C virus; PWID, people who inject drugs; SQ, status quo; SVR, sustained virological response.

Univariate sensitivity analyses on the EL scenario taken from perspective C show that the year by which the net economic benefit becomes positive (i.e., year by which the EL scenario becomes cost-saving) is robust to varying assumptions on the DAA medication costs, discount rate, utility weights, employment rates, and productivity measures for different levels of HCV disease (Fig 3). The largest effect is seen when assuming that the cost of a productive life year lost is based on median income rather than per-capita GDP (US$603.91 instead of US$1,443.63), which delays the year that HCV elimination becomes cost-saving to 2034 (95% UI 2033 to 2037). Conversely, assuming greater integration of HCV screening into existing services (two-thirds instead of one-third) and achieving the HCV elimination target sooner (by 2025 instead of 2030), results in the EL scenario becoming cost-saving earlier, by 2029 (95% UI 2028 to 2030) and 2029 (95% UI 2028 to 2031), respectively (Fig 3 and Table J in S1 Text).

## Discussion

In this analysis, we used dynamic HCV transmission modelling to show that achieving HCV elimination can bring about substantial societal health and economic benefits. Scaling up testing and treatment in Pakistan to reach the WHO target of reducing HCV incidence by 80% could prevent 333,000 HCV-related deaths and avert 5.57 million DALYs over 2018 to 2030. If HCV screening could be partially integrated into existing health services, this elimination initiative could cost the health system an additional US$1.45 billion in direct costs (testing, treatment, disease management) above the cost of existing initiatives over 2018 to 2030. However, US$1.30 billion could be saved over the same period through improvements in productivity from reduced absenteeism, presenteeism, and premature deaths due to HCV. This would make the investment highly cost-effective (ICER: US$29 per DALY averted) by 2030 and cost-saving shortly after, with a net economic benefit of US$9.10 billion by 2050. Our findings provide evidence that can help decision-makers balance short-term investments in HCV against investment in other priority areas, by quantifying the amount and time frame over which resulting health and economic benefits will occur.

Our analyses suggest that when services are integrated into existing health systems, costs are reduced and economic benefits are improved. This is not surprising and underpins the rationale for the universal health coverage (UHC) target of the United Nations Sustainable Development Goals (UN SDGs), suggesting that if UHC can be achieved, it will provide a means to realise the economic benefits of managing other health issues. Integration of HCV care into the PHCS network could have broader benefits including raising awareness among PHCS staff of how to reduce HCV transmission risks through reducing unnecessary medical injections and implementing improved infection control practices. Furthermore, integration at the PHCS level could also raise general community awareness of HCV, facilitate the development of community prevention programmes (e.g., safer barbering as has been observed in Egypt), and improve engagement in community testing programmes.

Our findings suggest that under the SQ scenario, economic productivity losses associated with HCV are likely to be approximately 1.5 times the direct healthcare management costs associated with HCV. New evidence is emerging on the indirect social and economic impacts of HCV [26,27,35–37], which is supported by recent work highlighting that countries may be substantially underestimating how much HCV is costing their economy [29,38]. This may be particularly important for LMICs where the direct costs associated with HCV epidemics may be minimal due to limited health services capacity to manage HCV-related disease. In Pakistan, this effect may be amplified because the burden of infection disproportionately impacts on people with poorer health or lower socioeconomic status [19], as well as marginalised groups such as PWID [39–41] and prisoners [42,43], who may have limited opportunities to access adequate healthcare services.

When only taking the health provider perspective (perspective A) and ignoring productivity gains, HCV elimination was estimated to cost US$417 per DALY averted by 2030, reducing to US$181 per DALY averted when productivity gains are included (perspective B) and US$29 per DALY averted with partial integration of direct costs (perspective C). These cost-effectiveness estimates are useful for comparison with other health interventions, as well as the “health opportunity cost” for Pakistan, which is a benchmark for assessing the cost-effectiveness of new healthcare interventions; estimated as 9% to 12% of GDP per capita (US$148 to US$198 in 2018 for Pakistan) [34]. Against this health opportunity cost threshold, HCV elimination is likely to be cost-effective by 2030 and cost-saving soon thereafter if we include productivity gains.

Another consideration is affordability of HCV elimination for Pakistan, with elimination and partial integration estimated to require an additional US$1.45 billion in direct costs. Given finite resources, countries need to decide which interventions are provided as part of UHC, commonly known as its country-specific health benefits package. The Disease Control Priorities (DCP) project is a guide to assist LMICs in developing their health benefits packages. Pakistan has recently reviewed its DCPs and included HCV testing and treatment in its list of essential health services. Moreover, the Government of Pakistan has pledged to increase the health sector’s allocation of funding to 3% of GDP by 2025 [44]. This means that HCV elimination could be financed through Pakistan’s expanded and revised country-specific health benefits package, provided sufficient evidence is available to document why HCV should be prioritised over other health issues. The evidence provided in this study has been highly relevant to this, providing an objective means to assist decision-makers in the prioritisation of funds between HCV and other health issues under consideration in the updated DCPs.

### Strengths and limitations

We have previously estimated the total direct costs of screening, treatment, and healthcare management required to eliminate HCV in Pakistan [14]. Our analysis presented here builds on the previous modelling, with the main strength of this analysis being to estimate the additional societal health and economic benefits of achieving HCV elimination in a high-burden LMIC in terms of gains in HRQoL and productivity, and that this is the first to consider the effect of integrating HCV screening into existing services. For Pakistan, this provides crucial information to policymakers as discussed above.

Despite this, limitations exist, and we explored the impact of these limitations in our sensitivity analyses. Firstly, the possibility of partially integrating the testing pathway into existing health services was an important assumption that reduced costs considerably. Given that simplified HCV testing and treatment protocols are already being implemented by hospitals in Pakistan [45], it is reasonable to assume these programmes could be partially integrated into the existing PHCS network. However, it is uncertain what level of integration is possible and, although future studies need to consider this, our sensitivity analyses suggest that even with low levels of integration (one-sixth), our EL scenario would still become cost-saving by 2032. Moreover, the costs of testing and treatment (e.g., DAA medication costs) are dynamic, with specific costs varying across the country and over time. Nevertheless, our sensitivity analyses suggest that these variations should not affect our conclusions. Secondly, our productivity estimates used national employment rates and average wage levels based on per-capita GDP to calculate economic productivity gains. In reality, individuals with HCV may be more likely from lower socioeconomic communities with greater unemployment and/or lower wages, and so we have possibly overestimated the productivity gains from HCV elimination. Conversely, we did not account for HCV-related productivity losses resulting from unpaid work (e.g., domestic work, childcare) or the unofficial economy, so we have potentially underestimated productivity gains. However, our sensitivity analyses on varying the cost of a productive life year lost, including using median income instead of per-capita GDP, suggest that assuming lower wages has a moderate, but still limited, effect on delaying the year that HCV elimination becomes cost-saving in Pakistan, emphasising the importance of accurately capturing how improvements to productivity are valued and the benefits of curing HCV infection to the economy. Thirdly, our estimates for the effect of HCV infection on productivity came from high-income countries, and so could differ in Pakistan. Although we incorporated uncertainty in our productivity estimates to account for these issues, and our results were robust despite this, it also highlights the need for data on this for Pakistan and other LMICs. Fourthly, to quantify the economic benefits, we have undertaken a cost-effectiveness analysis rather than a cost–benefit analysis, and so we did not convert health units (DALYs) into economic units. Calculating cost–benefit ratios could provide further evidence on the benefits of HCV elimination in Pakistan. Fifthly, with the impetus of HCV elimination programmes worldwide and, in particular, the massive HCV intervention scale-up campaign in Egypt, where they screened 50 million persons in a 7-month period [13], a similar national scale-up initiative in Pakistan could lead to HCV elimination being achieved earlier than 2030, which would entail higher up-front costs, but also incur greater potential long-term health and economic benefits from averted infections, liver disease, and HCV-related deaths in the future. Our sensitivity analyses support this hypothesis. However, more detailed studies are needed to investigate the feasibility of achieving the HCV elimination target sooner in Pakistan, and how it would affect the year in which HCV elimination becomes cost-effective and, ultimately, cost-saving. Lastly, there is no data on Pakistan-specific utility weights for HCV-related disease, and so we used generic values taken from the GBD 2017 study [24,25], which do not include disability weights for all HCV infection disease stages [46].

### Comparison with other studies

There is increasing evidence that scaling up HCV treatment is highly cost-effective and potentially cost-saving in a number of settings. Subnational studies in various LMICs have shown DAA treatment of HCV-diagnosed patients to be cost-effective in Myanmar [47] and cost-saving in India, Thailand, and South Africa, but they did not include screening costs [48]. Other subnational studies in Egypt [49], Cambodia [6], and Pakistan [21] have shown that screening and DAA treatment can be cost-effective or possibly cost-saving when compared to no screening and treatment. Besides these and our previous analyses [14], only one other analysis has evaluated the costs of undertaking a national scale-up of treatment to achieve the WHO HCV elimination targets. This prior study for Pakistan [50] included costs for diagnostics and treatment but did not include any costs for retesting individuals who previously tested negative and did not include any risk of reinfection, which are important in settings like Pakistan where HCV transmission is ongoing. These are included in our analyses. Additionally, all previous modelling and economic analyses of HCV treatment have only focussed on the health-related effects, benefits, and costs of HCV infection and treatment. However, as well as reducing morbidity and onwards transmission, empirical data from other settings have shown that curing people of HCV can achieve substantial economic gains through increased productivity [51]. Unfortunately, no primary data exist on this for LMIC settings. Lastly, a recent global modelling analysis estimated that reaching the global HCV elimination targets could generate a net US$22.7 billion economic benefit by 2030, primarily as a result of improvements in workforce productivity [29,38]. This model was conducted at the WHO region level for advocacy to highlight how the true costs of HCV burden (and gains from elimination) are being underestimated by only considering the health system perspective, but it did not include country-specific empirical cost estimates and detailed country-level epidemic calibrations, with our analysis undertaking more nuanced modelling to guide decision-making in Pakistan.

## Conclusions

Pakistan is well placed to capitalise on the scientific advancement in HCV treatment through leading the world in achieving HCV elimination. Given the high HCV burden in Pakistan and its potential catastrophic health impact, HCV elimination should be considered as a disease priority to enable Pakistan to fully realise the health and societal benefits of low-cost DAAs. HCV has already been included in Pakistan’s health benefits package for UHC, but now to achieve elimination, greater efforts are needed to implement and expand testing and treatment at scale, something that the Government of Pakistan has committed to on World Hepatitis Day 2019 [52]. This will ensure not only that life-saving HCV cure is available to all, but will achieve substantial societal health and economic benefits for Pakistan.

## Supporting information

S1 TextSupporting information.Additional details of the model and methods; input parameters and calibration data; additional results tables and figures; CHEERS checklist; and references. **Methods A. Productivity gains from people cured of HCV. Methods B. Productivity gains from averted deaths. Fig A. Simplified HCV screening and treatment model schematic.** The full HCV transmission model schematic including demographic and behavioural compartments, disease progression stages, HCV infection and transmission dynamics, and complete screening and treatment cascade has been shown previously. **Fig B. Schematic of productivity model.** Parameters used in the productivity model are in Table 2, with specific reference to † and ‡. ^§^Treatment rates may be different among PWID and cirrhotic patients, hence we allow $p_{PWID}\neq {\hat{p}}_{PWID}$ and $C\neq \hat{C}$. **Fig C. Estimated direct annual costs of testing, treatment, and healthcare management for the SQ and EL scenarios.** Model projections showing the estimated direct annual costs of testing, treatment, and healthcare management for the SQ and EL scenarios. The direct annual cost of elimination differs depending on whether testing is integrated or not. All costs are in 2018 US$ and discounted at 3.5% per annum; healthcare costs applied to all liver disease states pre- and post-cure; staffing costs applied to all testing and treatment interactions; one-third of initial screening not incurring staffing costs and reduced HCV RNA testing kit cost are assumed in the EL scenario with partial integration. The solid line and shading indicate the median and 95% UIs across 1,151 model fits. **Fig D. Estimated cost per DALY averted for the EL scenario compared to SQ.** Estimated cost per DALY averted for the EL scenario compared to SQ over different time horizons, from each of the economic perspectives. All costs and DALYs include discounting at 3.5% per annum, with costs in 2018 US$. The solid line and shading indicate the median and 95% UIs across 1,151 model fits. **Fig E. Univariate sensitivity analyses on overall net economic benefit by 2030 for the EL scenario.** For each sensitivity analysis scenario, the overall net economic benefit by 2030 for HCV elimination is taken from economic perspective C, compared to SQ. The dashed vertical line indicates the threshold where HCV elimination becomes cost-saving, i.e., there is a positive net economic benefit by 2030. The bars show the median across 1,151 model runs for the various sensitivity analyses. **Fig F. Heat map showing correlation coefficients between parameters across final baseline model fits.** Refer to S1 Table for the symbols corresponding to each of the model parameters. Note that baseline model parameters that are point estimates are not shown. These include the ageing parameters (***η*_1_, *η*_2_**), the age-specific death rates for the young and young adult categories (***μ*_1,*g*_, *μ*_2,*g*_**), and the relative risk of progression from DC to HCC if SVR (***ϵ_DH_***), which is assumed to be unity (S1 Table). In the heat map shown, between any pair of parameters, a correlation coefficient of “0” implies that no correlation is present, while a “1” or “-1” suggests a perfect positive or negative linear correlation, respectively. The age-specific death rate parameter ***μ*_3,*g*_** was derived by fitting to population growth trends, so would be expected to be correlated to population growth rate as shown. All other parameter sets for the baseline model (*n =* 1,151 final model fits) do not appear to be strongly correlated to each other. **Table A. Baseline HCV transmission model parameters with associated uncertainty ranges.** Rates are per year. **Table B. Screening and treatment model parameters with associated uncertainty ranges.** Rates are per year. EL, elimination scenario; SQ, status quo. **Table C. Demographic and epidemiological data used to calibrate and fit the model. Table D. Annual pre-intervention treatment numbers by province and in total. Table E. Model projections of the HCV-related morbidity and mortality for the SQ and EL scenarios over 2018–2030 or over 2018–2050.** DALYs are discounted at a rate of 3.5% per annum. The values represent the median and 95% UIs across 1,151 model fits. **Table F. Breakdown of absolute cost estimates for the economic components of the SQ and EL scenarios taking 3 different economic perspectives (A, B, and C).** Total costs, combined and split by direct and indirect costs, are determined over 2018–2030 and over 2018–2050. Costs are discounted at a rate of 3.5% per annum and are presented in 2018 US$. The values represent the median and 95% UIs across 1,151 model fits. **Table G. A summary of the incremental differences in the costs, overall as well as by direct costs and indirect costs, over 2018–2030 and 2018–2050 between the SQ scenario and the EL scenario from each of the 3 economic perspectives (A, B, and C).** Costs are discounted at a rate of 3.5% per annum and are presented in 2018 US$. The values represent the median and 95% UIs across 1,151 model fits. **Table H. ICERs for the modelled EL scenarios over 2018–2050 for 3 economic perspectives.** Costs and DALYs are discounted at 3.5% per annum, with costs presented in 2018 US$. Perspective A includes direct costs only (costs for testing, treatment, and healthcare management). Perspective B includes direct costs (perspective A) plus productivity costs. Perspective C includes partially integrated direct costs and productivity costs. The values represent the median and 95% UIs across 1,151 model fits. **Table I. Details of univariate sensitivity analyses scenarios investigated. Table J. Univariate sensitivity analyses for the EL scenario from economic perspective C.** Net economic benefit at 2030 is the negative of the total sum of direct and indirect costs over 2018–2030, with positive values indicating a net monetary gain (bolded entries) and negative values indicating a net monetary loss (see Fig 2B for net economic benefit over time at baseline). The baseline model projections using economic perspective C is shaded. Costs and DALYs are discounted at a rate of 3.5% per annum, with costs presented in 2018 US$. The values represent the median and 95% UIs across 1,151 model fits. **Checklist A. Consolidated Health Economic Evaluation Reporting Standards (CHEERS) checklist**. DALY, disability-adjusted life year; DC, decompensated cirrhosis; EL, elimination; HCC, hepatocellular carcinoma; HCV, hepatitis C virus; ICER, incremental cost-effectiveness ratio; PWID, people who inject drugs; SQ, status quo; SVR, sustained virological response; UI, uncertainty interval.(PDF)

## Abbreviations

Ab: antibody
DAA: direct-acting antiviral
DALY: disability-adjusted life year
DC: decompensated cirrhosis
DCP: Disease Control Priorities
EL: elimination
HCC: hepatocellular carcinoma
HCV: hepatitis C virus
HRQoL: health-related quality of life
ICER: incremental cost-effectiveness ratio
LMIC: low- and middle-income country
LTFU: lost to follow-up
MSF: Médecins Sans Frontières
PHCS: primary healthcare services
PWID: people who inject drugs
SQ: status quo
SVR: sustained virological response
UHC: universal health coverage
UI: uncertainty interval
UN SDG: United Nations Sustainable Development Goal
WHO: World Health Organization
WTP: willingness-to-pay
