# Correction: Health and economic benefits of achieving hepatitis C virus elimination in Pakistan: A modelling study and economic analysis

**DOI:** 10.1371/journal.pmed.1004423

**Published:** 2024-06-25

**Authors:** Aaron G. Lim, Nick Scott, Josephine G. Walker, Saeed Hamid, Margaret Hellard, Peter Vickerman

The second sentence of the Acknowledgments is incomplete. The complete sentence reads: AGL, JW, and PV also acknowledge funding from the Wellcome Trust under grant number 220866/Z/20/Z.
