# Supplementary material for: Health and economic benefits of achieving hepatitis C virus elimination in Pakistan: A modelling study and economic analysis
Source: PLoS Med. 2021 Oct 19;18(10):e1003818. doi: 10.1371/journal.pmed.1003818 (PMC8525773; doi:10.1371/journal.pmed.1003818)
Supplement: S1 Text — Additional details of the model and methods; input parameters and calibration data; additional results tables and figures; CHEERS checklist; and references. Methods A. Productivity gains from people cured of HCV. Methods B. Productivity gains from averted deaths. Fig A. Simplified HCV screening and treatment model schematic. The full HCV transmission model schematic including demographic and behavioural compartments, disease progression stages, HCV infection and transmission dynamics, and complete screening and treatment cascade has been shown previously. Fig B. Schematic of productivity model. Parameters used in the productivity model are in Table 2, with specific reference to † and ‡. §Treatment rates may be different among PWID and cirrhotic patients, hence we allow pPWID≠p^PWID and C≠C^. Fig C. Estimated direct annual costs of testing, treatment, and healthcare management for the SQ and EL scenarios. Model projections showing the estimated direct annual costs of testing, treatment, and healthcare management for the SQ and EL scenarios. The direct annual cost of elimination differs depending on whether testing is integrated or not. All costs are in 2018 US$ and discounted at 3.5% per annum; healthcare costs applied to all liver disease states pre- and post-cure; staffing costs applied to all testing and treatment interactions; one-third of initial screening not incurring staffing costs and reduced HCV RNA testing kit cost are assumed in the EL scenario with partial integration. The solid line and shading indicate the median and 95% UIs across 1,151 model fits. Fig D. Estimated cost per DALY averted for the EL scenario compared to SQ. Estimated cost per DALY averted for the EL scenario compared to SQ over different time horizons, from each of the economic perspectives. All costs and DALYs include discounting at 3.5% per annum, with costs in 2018 US$. The solid line and shading indicate the median and 95% UIs across 1,151 model fits. Fig E. Univariate sensitivity a [file pmed.1003818.s001.pdf]

S1 TEXT. SUPPORTING INFORMATION

Health and economic benefits of achieving hepatitis C virus elimination in Pakistan: A modelling study and economic analysis

Authors:
Aaron G Lim[1,†,*], DPhil
Nick Scott[2,†], PhD
Josephine G Walker[1], PhD
Saeed Hamid[3], MBBS, FRCP
Margaret Hellard[2], PhD
Peter Vickerman[1], DPhil

[1]Population Health Sciences, Bristol Medical School, University of Bristol, UK
[2]Burnet Institute, Melbourne, Australia
[3]Aga Khan University, Karachi, Pakistan
†Joint first author
*Corresponding author: aaron.lim@bristol.ac.uk

Table of Contents

SUPPLEMENTARY METHODS AND FIGURES 3

Fig A. Simplified HCV screening and treatment model schematic. 3
Methods A. Productivity gains from people cured of hepatitis C virus. 4
Fig B. Schematic of productivity model. 4
Methods B. Productivity gains from averted deaths. 4
Fig C. Estimated direct annual costs of testing, treatment, and healthcare management for the Status Quo and Elimination scenarios. 5
Fig D. Estimated cost per disability-adjusted life year (DALY) averted for the elimination scenario compared to Status Quo. 6
Fig E. Univariate sensitivity analyses on overall net economic benefit by 2030 for the elimination scenario. 7
Fig F. Heat map showing correlation coefficients between parameters across final baseline model fits. 8

SUPPLEMENTARY TABLES 9

Table A. Baseline HCV transmission model parameters with associated uncertainty ranges. 9
Table B. Screening and treatment model parameters with associated uncertainty ranges. 10
Table C. Demographic and epidemiological data used to calibrate and fit the model. 11
Table D. Annual pre-intervention treatment numbers by province and in total. 12
Table E. Model projections of the HCV-related morbidity and mortality for the status-quo (SQ) and elimination (EL) scenarios over 2018-2030 or over 2018-2050. 13
Table F. Breakdown of absolute cost estimates for the economic components of the status quo (SQ) and elimination (EL) scenarios taking three different economic perspectives (A, B, and C). 14
Table G. A summary of the incremental differences in the costs, overall as well as by direct costs and indirect costs, over 2018-2030 and 2018-2050 between the status quo scenario (SQ) and the elimination scenario (EL) from each of the three economic perspectives (A, B, and C). 15
Table H. Incremental cost-effectiveness ratios (ICERs) for the modelled elimination scenarios over 2018-2050 for three economic perspectives. 16
Table I. Details of univariate sensitivity analyses scenarios investigated. 17
Table J. Univariate sensitivity analyses for the elimination scenario from economic perspective C. 18

OTHER SUPPLEMENTARY MATERIALS 19

Checklist A. Consolidated Health Economic Evaluation Reporting Standards (CHEERS) checklist.[31] 19

SUPPLEMENTARY REFERENCES 22

Supplementary Methods and Figures

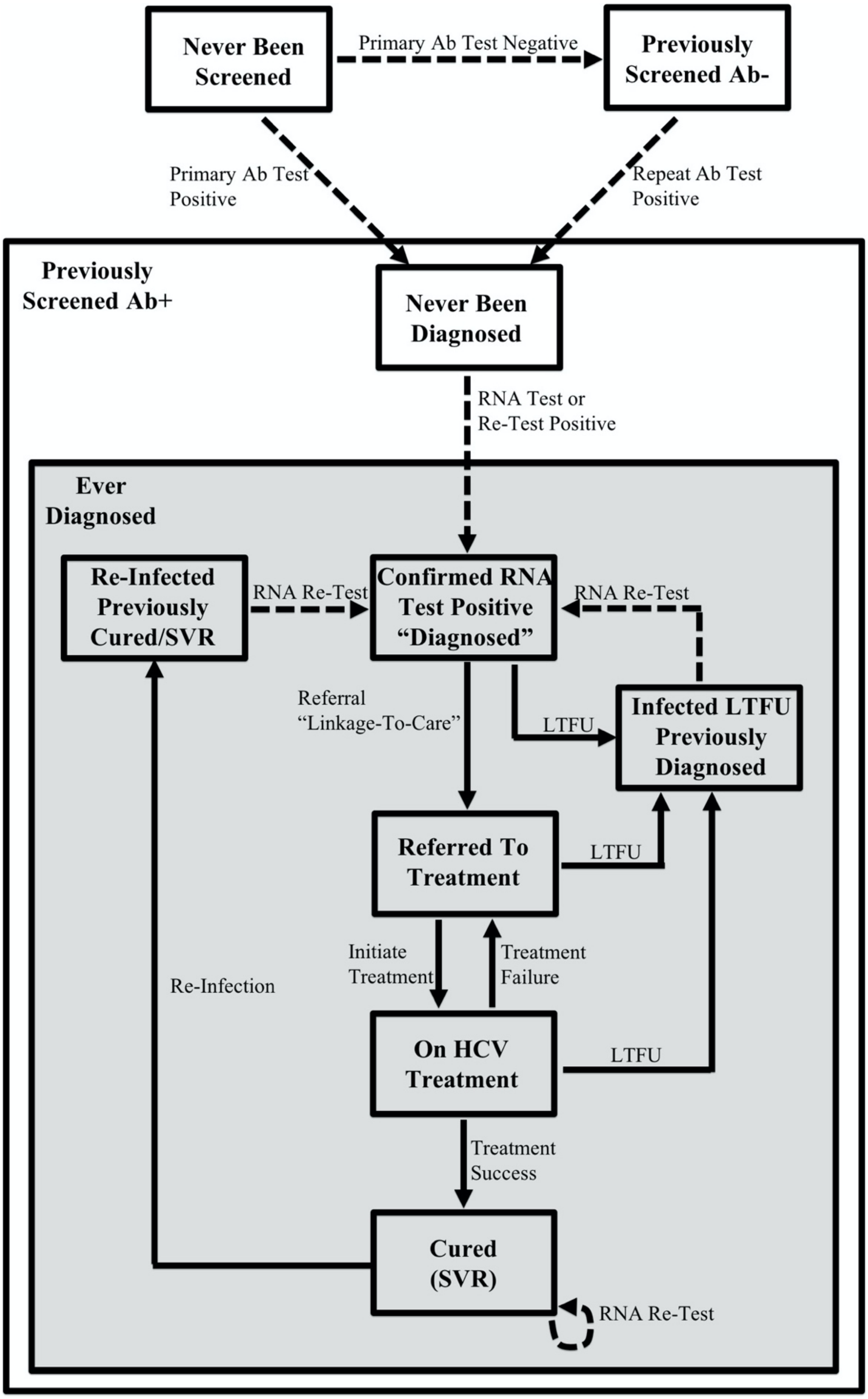

Fig A. Simplified HCV screening and treatment model schematic.
The full HCV transmission model schematic including demographic and behavioural compartments, disease progression stages, HCV infection and transmission dynamics, and complete screening and treatment cascade has been shown previously.[1]

Methods A. Productivity gains from people cured of hepatitis C virus.
An independent mathematical model was used to capture hepatitis C-attributable productivity losses from absenteeism (due to a reduced workforce or from individuals working reduced hours), and presenteeism (where individuals are less productive at work due to their illness) (Fig B). The model accounted for differential employment opportunities among PWID, as well as differential productivity and treatment uptake by cirrhosis status. The human capital approach[2] was used to estimate years of potential productive life lost, which were converted to economic outcomes using population-weighted average per capita gross domestic product (GDP). Total productivity losses were compared between the Status Quo and Elimination scenarios to determine economic gains. Parameters and sources are provided in Table 1.

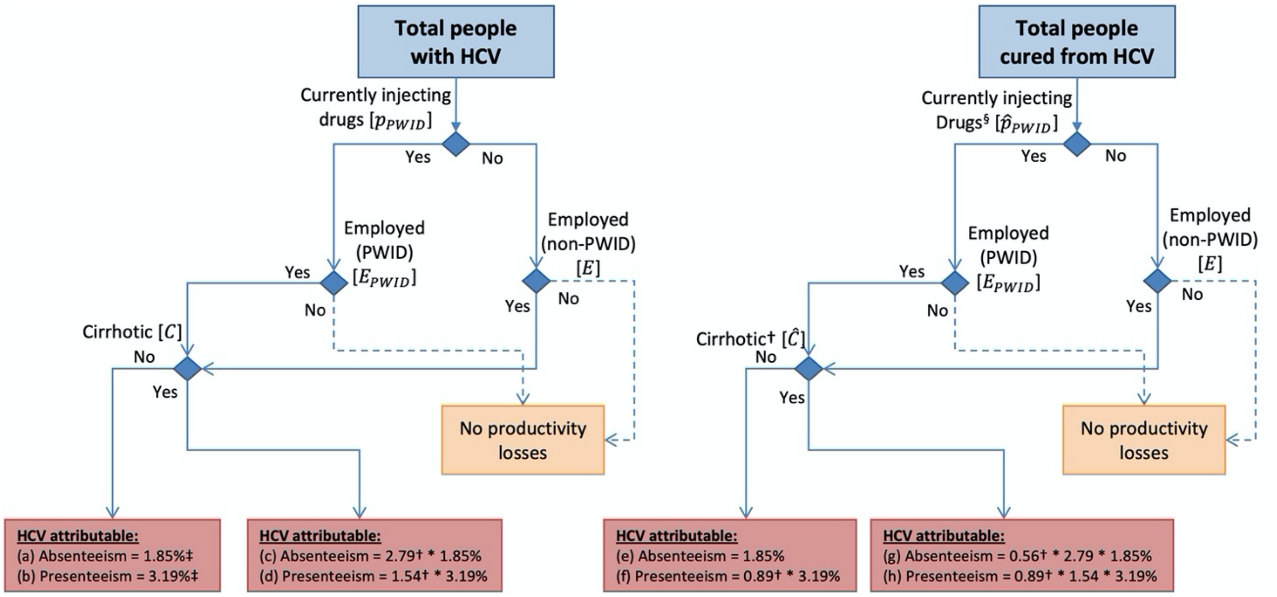

Fig B. Schematic of productivity model.
Parameters used in the productivity model are in Table 2, with specific reference to †[3] and ‡[4]. §Treatment rates may be different among PWID and cirrhotic patients, hence we allow $p_{PWID} \neq \hat{p}_{PWID}$ and $C \neq \hat{C}$.

Methods B. Productivity gains from averted deaths.
Productivity gains from deaths averted in the Status Quo (SQ) and Elimination (EL) scenarios were included. For both of these treatment scenarios, we first calculated the total number of deaths averted in a given year using the baseline dynamic model of HCV in Pakistan. However, a disproportionate amount of HCV-related deaths are estimated to occur among older age groups (Table 2 shows the estimated 2016 age distribution of HCV-related deaths for according to the WHO[5]), and therefore only a fraction of these averted deaths were assumed to result in years of productive life gained. For each year in the projection timeframes (2018-2030 or 2018-2050), the productive life gained from deaths averted in that year were calculated by assuming:

(i) The fraction of averted deaths among 60+ age category did not produce additional years of productivity.
(ii) Of the fraction of averted deaths among the 50-59 age category:
   - All of them contributed an additional year of productivity in the year they occurred;
   - 8/9th of these deaths contributed an additional year of productivity the year after they occurred (approximating 1/9th of this age band entering non-productive life at 60 years)
   - 7/9th of these deaths contributed an additional year of productivity two years after they occurred;
   - And so on, with the fraction of deaths averted from this age category contributing decreasing productivity gains for the next 9 years, before no longer producing additional productive years.
(iii) Of the fraction of averted deaths among the 30-49 age category, the methodology above was used to attribute their ongoing productive years following the year that their death was prevented.

Years of productive life lost due to premature death were converted to economic outcomes using population-weighted average per capita GDP. Future economic productivity gains were discounted at 3.5%.

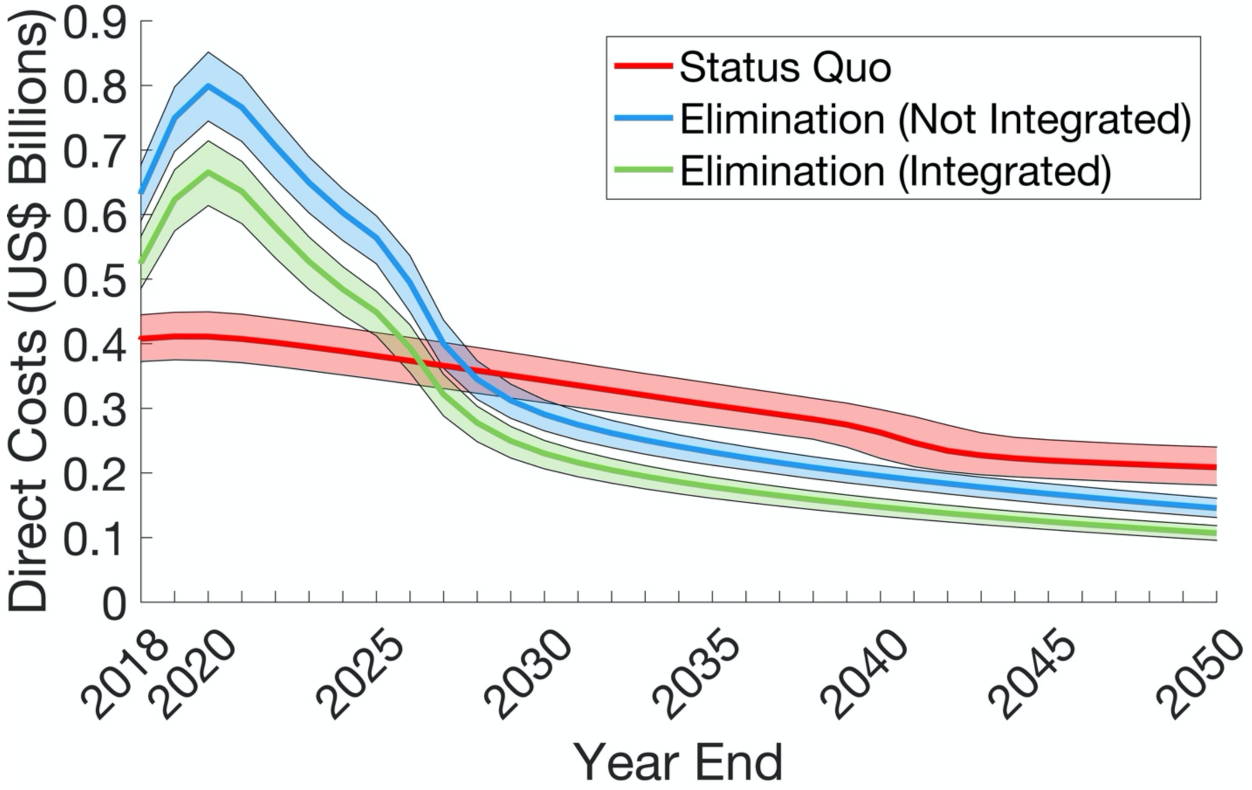

Fig C. Estimated direct annual costs of testing, treatment, and healthcare management for the Status Quo and Elimination scenarios.

Model projections showing the estimated direct annual costs of testing, treatment, and healthcare management for the Status Quo and Elimination scenarios. The direct annual cost of elimination differs depending on whether testing is integrated or not. All costs are in 2018 US$ and discounted at 3.5% per annum; healthcare costs applied to all liver disease states pre- and post-cure; staffing costs applied to all testing and treatment interactions; one-third of initial screening not incurring staffing costs and reduced HCV RNA testing kit cost are assumed in the elimination scenario with partial integration. The solid line and shading indicate the median and 95% uncertainty intervals (UI) across 1,151 model fits.

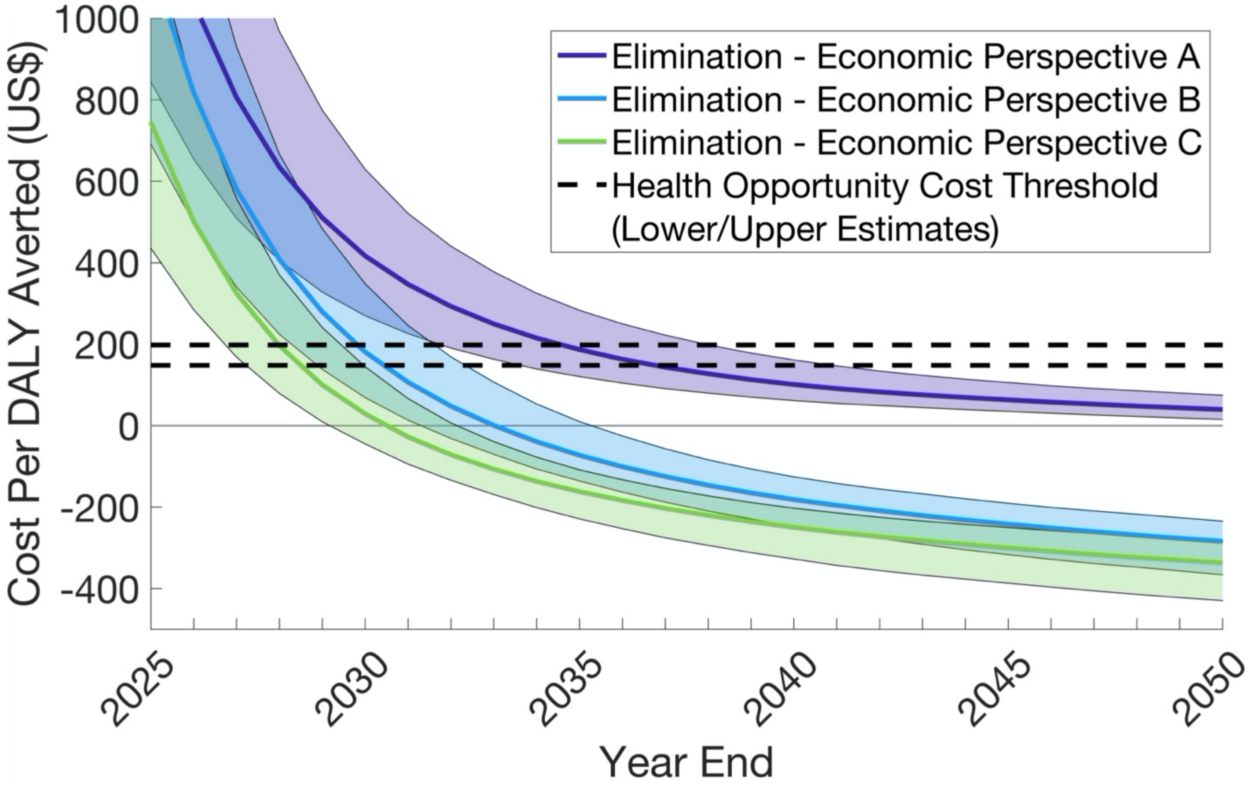

Fig D. Estimated cost per disability-adjusted life year (DALY) averted for the elimination scenario compared to Status Quo.

Estimated cost per disability-adjusted life year (DALY) averted for the elimination scenario compared to Status Quo over different time horizons, from each of the economic perspectives. All costs and DALYs include discounting at 3.5% per annum, with costs in 2018 US$. The solid line and shading indicate the median and 95% uncertainty intervals (UI) across 1,151 model fits.

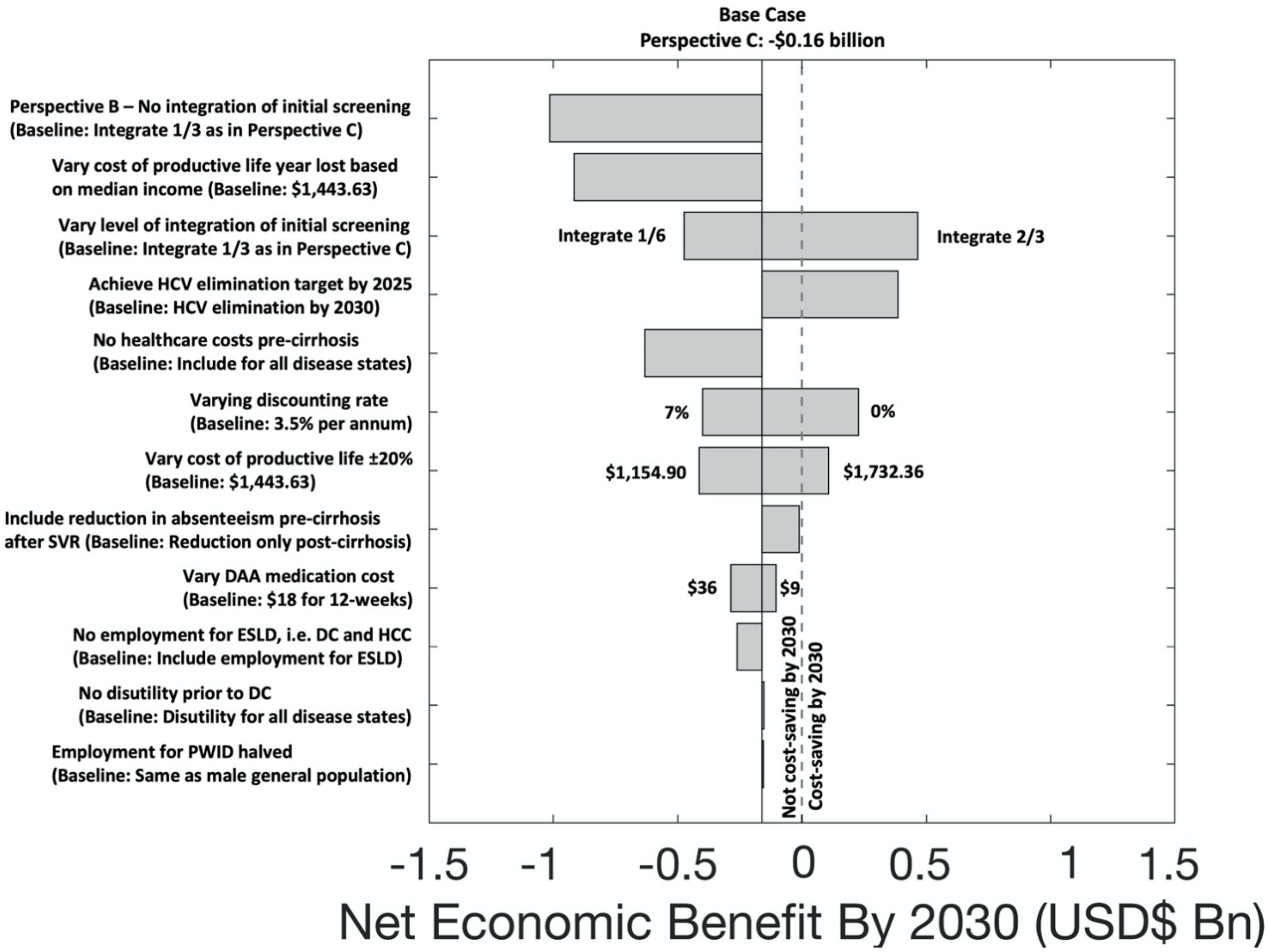

Fig E. Univariate sensitivity analyses on overall net economic benefit by 2030 for the elimination scenario.

For each sensitivity analysis scenario, the overall net economic benefit by 2030 for HCV elimination is taken from economic perspective C, compared to Status Quo The dashed vertical line indicates the threshold where HCV elimination becomes cost-saving, i.e. there is a positive net economic benefit by 2030. The bars show the median across 1,151 model runs for the various sensitivity analyses.

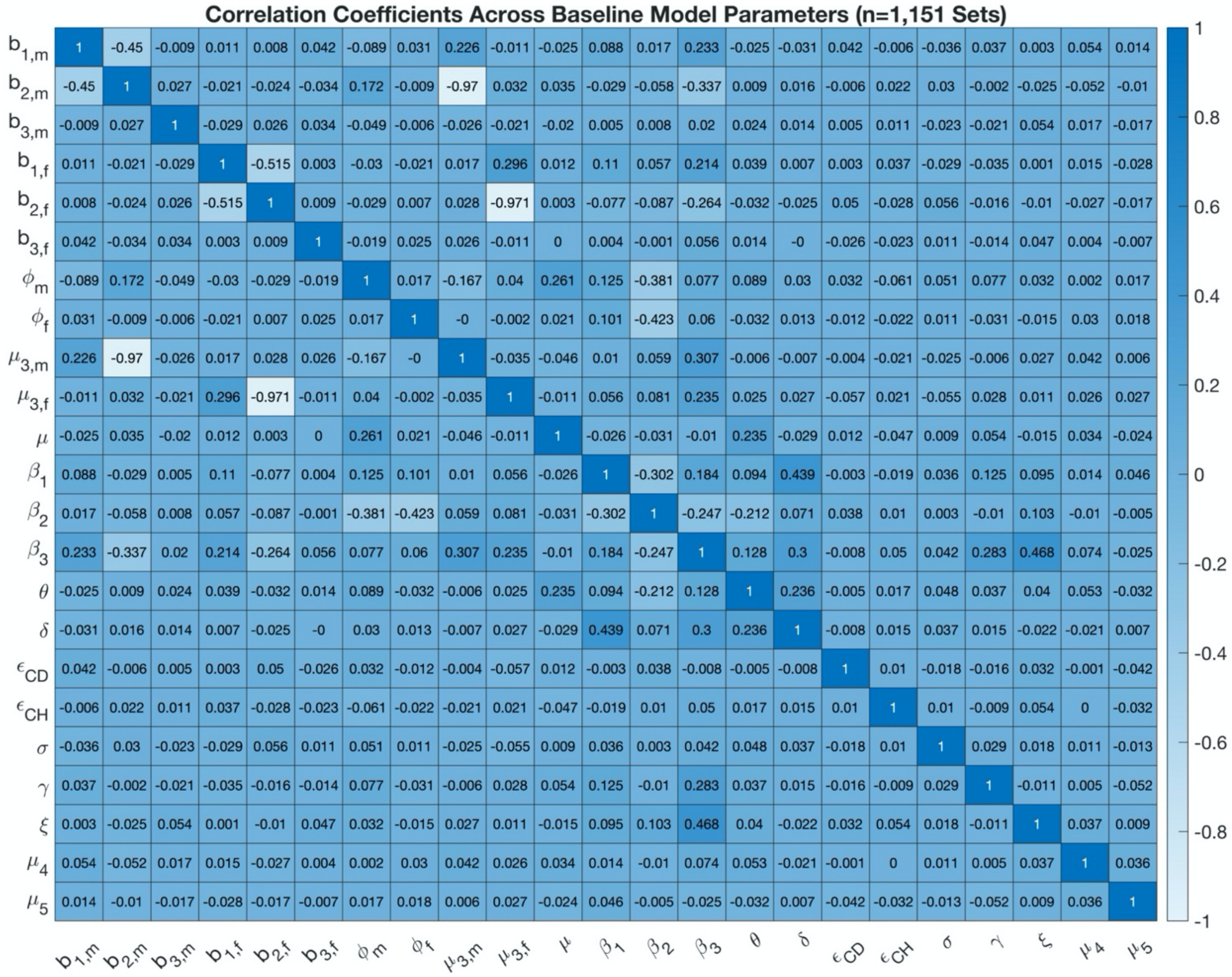

Fig F. Heat map showing correlation coefficients between parameters across final baseline model fits. Refer to Table A for the symbols corresponding to each of the model parameters. Note that baseline model parameters that are point estimates are not shown. These include the ageing parameters (η_1, η_2), the age-specific death rates for the young and young adult categories $(\mu_{1,g}, \mu_{2,g})$, and the relative risk of progression from DC to HCC if SVR (ϵ_{DH}) which is assumed to be unity (Table A). In the heatmap shown, between any pair of parameters, a correlation coefficient of '0' implies no correlation is present, while a '1' or '-1' suggests a perfect positive or negative linear correlation, respectively. The age-specific death rate parameter $\mu_{3,g}$ was derived by fitting to population growth trends, so would be expected to be correlated to population growth rate as shown. All other parameter sets for the baseline model (n=1,151 final model fits) do not appear to be strongly correlated to each other.

Supplementary Tables

Table A. Baseline HCV transmission model parameters with associated uncertainty ranges.
Rates are per year.

Parameter	Symbol	Baseline value or fitted range when stated [Uncertainty Distribution/Range]	Source
**Demographic Parameters**			
Average population growth rate per annum Δ	$b_{1,g}$<br>$b_{2,g}$<br>$b_{3,g}$	Pre-2000: Fitted 2.76% [2.53 %-2.99%]<br>Interim 2000-2015: Fitted 1.92% [1.54-2.31%]<br>Post-2015: [Uniform 1.35-2.08%]	[6-8]
Rate of ageing from Young to Young Adult	η_1	1/20	Based on average duration of 20 years in 0-19 age group
Rate of ageing from Young Adult to Adult	η_2	1/10	Based on average duration of 10 years in 20-29 age group
Proportion of Young Adults who initiate injecting drug use	ϕ_g	Fitted values: Male: 0.032 [0.026-0.039], Female: 0.009 [0.0004-0.017]	Calibrated to fit PWID proportions given in Table C[9]
Average mortality rate for each age group	$\mu_{1,g}$<br>$\mu_{2,g}$<br>$\mu_{3,g}$	1/56<br>1/41 Fitted: 0.024<br>Fitted values: Male: 0.023 [0.020-0.026]<br>Female: 0.020 [0.017-0.024]	Based on a life expectancy at birth estimate of 66 years in 2015[8], but also adjusted in model calibration
Additional drug-related mortality rate	μ	0.028 [Lognormal 0.017-0.039]	Based on estimates of drug-related mortality across Asia[10]
**Epidemic/Transmission Parameters**			
HCV transmission rate per susceptible in each age group (fitted values)	β_1<br>β_2<br>β_3	β_1 = 0.059 [0.052-0.066]<br>β_2 = 0.053 [0.023-0.085]<br>β_3 = 0.12 [0.10-0.14]	Fit to chronic prevalence in each age category in 2007[11] as given in Table C
Additional HCV transmission rate for injecting drug use	θ	Fit to data on HCV prevalence amongst PWID<br>Fitted values: 0.61 [0.51-0.74]	Fit to chronic prevalence in PWID[12,13] in 2012: 62.2% [55.5-68.8%]
Proportion of infections that spontaneously clear	δ	0.26 [Uniform 0.22-0.29]	[12]
**Disease Progression Parameters**			
Relative risk of progression from cirrhosis to decompensated if SVR	ϵ_{CD}	0.07 [Lognormal 95% CI 0.03, 0.20]	[14]
Relative risk of progression from cirrhosis to HCC if SVR	ϵ_{CH}	0.23 [Lognormal 95% CI 0.16, 0.35]	[14,15]
Relative risk of progression from decompensation to HCC if SVR	ϵ_{DH}	1.0	Assume same progression for both SVR and non-SVR
Relative risks of disease progression if infected by HCV genotype 3†	---	1.30 [Uniform 1.22-1.39] for chronic to cirrhosis, and cirrhosis to decompensation<br>1.80 [Uniform 1.60-2.03] for cirrhosis/decompensation to HCC	[16]
Transition probability (TP) of chronic HCV to cirrhosis†‡	σ	0.027 [Normal – mean = 0.027, std = 0.0008]	[17]
TP of compensated cirrhosis to decompensation†‡	γ	0.039 [Beta– $\alpha = 14.6, \beta = 360.2$]	[18-20]
TP of cirrhosis or decompensation to HCC†‡	ξ	0.014 [Beta– $\alpha = 1.9, \beta = 136.1$]	[18-20]
TP of additional mortality due to decompensation‡	μ_4	0.13 [Beta– $\alpha = 147.0, \beta = 984.0$]	[18-20]
TP of death due to HCC‡	μ_5	0.43 [Beta– $\alpha = 117.1, \beta = 155.2$]	[18-20]

Δ Baseline values for b for the pre-2000 and interim 2000-2015 growth rates are taken from the UN Department of Economic and Social Affairs, Population Division[8]; the projected post-2015 growth rate at baseline is obtained by averaging the point projections for the years 2015 to 2030 from the International Data Base, US Census Bureau[21]
†The transition probabilities listed here are calibrated to reflect the higher proportion of HCV genotype 3 in Pakistan, which is associated with an increased transition probability of disease progression.[16]
‡Transition probabilities have been converted to instantaneous rates for use in the model.

Table B. Screening and treatment model parameters with associated uncertainty ranges.
Rates are per year. SQ: Status Quo. E: Elimination scenario.

Parameter	Baseline value or fitted range when stated [Uncertainty Distribution/Range]	Source
**Screening Parameters**		
Primary Ab screening rate	SQ: 2.6-5.9%<br>E: 12.4%	[1]
Ab re-screening rate of SVR and previously screened uninfected	SQ: N/A<br>E: General population 20%, PWID 100%	[1]
Proportion of primary Ab-screened persons tested for HCV RNA	Set to 1	Assume that everyone who is tested Ab-positive, either from primary Ab screening or Ab re-screening, are subsequently tested for HCV RNA, i.e. there is no LTFU at this stage.[1]
Proportion of Ab re-screened persons tested for HCV RNA	Set to 1	
RNA re-screening of previously treated or previously diagnosed LTFU linked back to care	SQ: 0<br>E: General population 20%, PWID 100%	[1]
**Referral Parameters**		
Referral rate to treatment	SQ: 35-70%<br>E: 90%	[1]
**Treatment Parameters**		
Treatment rate per capita	Calibrated to historical treatment rate at baseline. From 2018, the value is set to 1.6094 so that approx. 80% of referred individuals will initiate treatment within the next year	Note: A rate r corresponds to a proportion $p = (1 - e^{-rt})$ transitioning by time t
Average duration on treatment	24-weeks for conventional treatment with IFN and RBV, which was the standard for treatment of HCV genotype 3 in Pakistan before 2016. Shortened to 12 weeks for pre-cirrhotic patients when DAA treatments were introduced from 2016 onwards; patients with cirrhosis or ESLD commence HCV treatment for 24 weeks	[22,23]
Proportion of individuals achieving SVR with IFN and RBV treatment	0.61 [Uniform 0.50-0.726]	[24-26]
Proportion of individuals achieving SVR with new DAA treatments	0.9 [Uniform 0.80-0.95]	[22,23,27]
**Lost to Follow Up (LTFU) Parameters**		
LTFU following diagnosis	Set to the proportion not referred to treatment	Assume that those who have been diagnosed and are not referred to treatment are LTFU.[1]
LTFU during referral	Set to 0	
LTFU during treatment	Set to 0	

Table C. Demographic and epidemiological data used to calibrate and fit the model.

Demographic and Epidemiological Data		Baseline Value [Uncertainty Distribution/Range]	Source
Total Population in 1960	Total	[Uniform 44,912,000–51,719,000]	[6-8]
	Male	[Uniform 24,058,000-27,704,304]	
	Female	[Uniform 20,854,000-24,014,696]	
Total Population in 2000	Total	[Uniform 138,250,000–152,429,036]	[6-8]
	Male	[Uniform 71,330,000-78,324,451]	
	Female	[Uniform 66,921,000-74,104,585]	
Total Population in 2015	Total	[Uniform 188,925,000–199,085,847]	[6-8]
	Male	[Uniform 97,052,000-102,231,058]	
	Female	[Uniform 91,873,000-96,854,789]	
Proportion in Each Age Group	0-19 years old	43.7%	[8]
	20-29 years old	19.3%	
	30+ years old	37.0%	
PWID size estimate	Whole population	0.24% [Uniform 0.18-0.30%]	UNODC 2013[9]
	Male	0.42% [Uniform 0.36-0.54%]	
	Female	0.006% [Uniform 0.0006-0.24%]	
HCV chronic prevalence in 2007 (estimated as 74% of antibody prevalence)	Overall	3.62% [3.45-3.79%]	[11], Estimated 95% binomial CI
	0-19 years old	1.50% [1.34-1.67%]	
	20-29 years old	3.20% [2.84-3.59%]	
	30+ years old	6.89% [6.50-7.30%]	
HCV chronic prevalence in PWID		62.16% [55.50-68.75%]	[13]
Projected change in HCV seroprevalence over 10 years		0.39% [-0.17 to 0.94%]	Meta-analysis on blood donor data trends in Pakistan from 1994 to 2014
Projected change in chronic HCV prevalence over 10 years		[Uniform -0.13 to 0.73%]	Assume full range of viraemic rate from spontaneous clearance[12]

Table D. Annual pre-intervention treatment numbers by province and in total.

Year	Punjab	Sindh	KPK	Baluchistan	Total Treatments Public Sector	Total Treatments Across All Sectors*
2005-2010	ND	ND	ND	ND	23,000	57,500
2011	ND	25,394	8,928	866	55,188[a]	137,970
2012	20,000	21,824	9,223	712	51,759	129,398
2013	20,000	28,221	6,212	731	55,164	137,910
2014	20,000	22,431	3,117	820	46,368	115,920
2015	34,500	21,847	3,837	900	61,084	152,710
2016[b]	-	-	-	-	-	152,710[b]
2017[b]	-	-	-	-	-	152,710[b]
**Total**						**1,036,828**

ND: No data available

*To estimate the total number of historical HCV treatments each year across both public and private sectors, a split of Public 40%, Private 60% was assumed. DAAs became available in the public sector from 2016 onwards.

[a]There were no data available for Punjab province in 2011, so it was assumed that 20,000 HCV patients were treated in 2011 under the Provincial Hepatitis Program, which is consistent with data from subsequent years 2012 to 2014.

[b]Data were not available for the pre-intervention years 2016 and 2017, so it was assumed that the total number of treatments nationally remained the same as in 2015.

Table E. Model projections of the HCV-related morbidity and mortality for the status-quo (SQ) and elimination (EL) scenarios over 2018-2030 or over 2018-2050.

DALYs are discounted at a rate of 3.5% per annum. The values represent the median and 95% uncertainty intervals across 1,151 model fits.

**2030 Estimates**		**Status Quo**	**Elimination**
**People living with hepatitis C in 2030 (millions)**	Total	8.99 (8.12 – 10.00)	1.21 (1.05 - 1.39)
	Averted	--	7.78 (7.03-8.66)
	% Reduction	--	86.5% (85.5-87.4%)
**Cumulative hepatitis C-related deaths 2018-2030**	Total	1,153,000 (811,000-1,678,000)	821,000 (589,000-1,105,000)
	Averted	--	333,000 (219,000-509,000)
	% Reduction	--	28.9% (25.2-33.1%)
**Total DALYs* 2018-2030 (millions)**	Total	24.06 (18.58-31.42)	18.53 (14.61-23.43)
	YLD	5.40 (3.79-7.09)	4.69 (3.21-6.21)
	YLL	18.63 (13.09-25.71)	13.78 (9.90-18.57)
	Averted	--	5.57 (3.80-8.22)
	% Reduction	--	23.2% (19.6-27.5%)

*Total DALYs = Years Lived with Disability (YLD) + Years of Life Lost (YLL)

**2050 Estimates**		**Status Quo**	**Elimination**
**People living with hepatitis C in 2050 (millions)**	Total	14.94 (12.05-17.25)	0.62 (0.47-0.88)
	Averted	--	14.31 (12.48-16.55)
	% Reduction	--	95.9% (94.3-96.6%)
**Cumulative hepatitis C-related deaths 2018-2050 (millions)**	Total	3.56 (2.48-5.00)	1.26 (0.89-1.72)
	Averted	--	2.31 (1.57-3.32)
	% Reduction	--	65.0% (60.8-67.8%)
**Total DALYs* 2018-2050 (millions)**	Total	52.30 (40.17-68.36)	25.76 (20.59-31.98)
	YLD	11.92 (8.28-15.79)	7.65 (5.11-10.29)
	YLL	40.12 (28.09-55.94)	17.97 (12.79-24.40)
	Averted	--	26.45 (19.31-36.70)
	% Reduction	--	50.9% (46.7-55.0%)

*Total DALYs = Years Lived with Disability (YLD) + Years of Life Lost (YLL)

Table F. Breakdown of absolute cost estimates for the economic components of the status quo (SQ) and elimination (EL) scenarios taking three different economic perspectives (A, B, and C).
Total costs, combined and split by direct and indirect costs, are determined over 2018-2030 and over 2018-2050. Costs are discounted at a rate of 3.5% per annum and are presented in 2018 US$. The values represent the median and 95% uncertainty intervals across 1,151 model fits.

Table F1. Modelled total cost estimates over 2018-2030. Amount in US$ billions.

		Status Quo (SQ)	Elimination (EL)
Perspective A	**Total Direct Costs**	$5.00 (4.53-5.48)	$7.32 (6.80-7.78)
	Screening	$1.49 (1.45-1.53)	$3.89 (3.76-4.02)
	Treatment	$0.23 (0.21-0.26)	$1.06 (0.96-1.18)
	Healthcare management	$3.27 (2.85-3.72)	$2.36 (2.00-2.71)
	**Indirect Costs**		
	Productivity	--	--
**Total Direct & Indirect Costs**		**$5.00 (4.53-5.48)**	**$7.32 (6.80-7.78)**
Perspective B	**Total Direct Costs**	$5.00 (4.53-5.48)	$7.32 (6.80-7.78)
	Screening	$1.49 (1.45-1.53)	$3.89 (3.76-4.02)
	Treatment	$0.23 (0.21-0.26)	$1.06 (0.96-1.18)
	Healthcare management	$3.27 (2.85-3.72)	$2.36 (2.00-2.71)
	**Indirect Costs**		
	Productivity	$7.11 (5.45-9.03)	$5.81 (4.47-7.33)
**Total Direct & Indirect Costs**		**$12.09 (10.31-14.19)**	**$13.12 (11.69-14.85)**
Perspective C	**Total Direct Costs**	$4.50 (4.04-4.97)	$5.97 (5.49-6.40)
	Screening	$1.01 (0.98-1.04)	$2.61 (2.52-2.70)
	Treatment	$0.22 (0.19-0.24)	$0.99 (0.89-1.10)
	Healthcare management	$3.27 (2.85-3.72)	$2.36 (2.00-2.71)
	**Indirect Costs**		
	Productivity	$7.11 (5.45-9.03)	$5.81 (4.47-7.33)
**Total Direct & Indirect Costs**		**$11.60 (9.82-13.68)**	**$11.77 (10.36-13.49)**

Table F2. Modelled total cost estimates 2018-2050. Amount in US$ billions.

		Status Quo (SQ)	Elimination (EL)
Perspective A	**Total Direct Costs**	$10.22 (9.16-11.33)	$11.28 (10.47-12.00)
	Screening	$2.72 (2.56-2.90)	$6.78 (6.45-7.10)
	Treatment	$0.36 (0.32-0.40)	$1.17 (1.05-1.31)
	Healthcare management	$7.13 (6.16-8.19)	$3.33 (2.77-3.83)
	**Indirect Costs**		
	Productivity	--	--
**Total Direct & Indirect Costs**		**$10.22 (9.16-11.33)**	**$11.28 (10.47-12.00)**
Perspective B	**Total Direct Costs**	$10.22 (9.16-11.33)	$11.28 (10.47-12.00)
	Screening	$2.72 (2.56-2.90)	$6.78 (6.45-7.10)
	Treatment	$0.36 (0.32-0.40)	$1.17 (1.05-1.31)
	Healthcare management	$7.13 (6.16-8.19)	$3.33 (2.77-3.83)
	**Indirect Costs**		
	Productivity	$19.73 (15.00-24.93)	$10.92 (8.48-13.65)
**Total Direct & Indirect Costs**		**$29.87 (24.95-35.58)**	**$22.21 (19.64-25.10)**
Perspective C	**Total Direct Costs**	$9.32 (8.28-10.39)	$8.97 (8.23-9.63)
	Screening	$1.85 (1.74-1.97)	$4.55 (4.33-4.77)
	Treatment	$0.34 (0.30-0.38)	$1.09 (0.97-1.22)
	Healthcare management	$7.13 (6.16-8.19)	$3.33 (2.77-3.83)
	**Indirect Costs**		
	Productivity	$19.73 (15.00-24.93)	$10.92 (8.48-13.65)
**Total Direct & Indirect Costs**		**$28.98 (24.09-34.69)**	**$19.90 (17.36-22.81)**

Table G. A summary of the incremental differences in the costs, overall as well as by direct costs and indirect costs, over 2018-2030 and 2018-2050 between the status quo scenario (SQ) and the elimination scenario (EL) from each of the three economic perspectives (A, B, and C).
Costs are discounted at a rate of 3.5% per annum and are presented in 2018 US$. The values represent the median and 95% uncertainty intervals across 1,151 model fits.

	**Incremental Costs (US$ Billions)**	
	**2030 Estimates**	**2050 Estimates**
**Perspective A**	**$2.31 (2.15 to 2.47) (direct costs only)**	**$1.06 (0.49 to 1.56) (direct costs only)**
**Perspective B**	**$1.01 (0.52 to 1.45), consisting of:**<br>$2.31 (2.15 to 2.47) in direct costs<br>$1.30 (0.94 to 1.72) in productivity gains	**$7.68 (5.13 to 10.58) in SAVINGS, consisting of:**<br>$1.06 (0.49 to 1.56) in direct costs<br>$8.76 (6.52 to 11.36) in productivity gains
**Perspective C**	**$0.16 (-0.33 to 0.59), consisting of:**<br>$1.45 (1.32 to 1.60) in direct costs<br>$1.30 (0.94 to 1.72) in productivity gains	**$9.10 (6.54 to 11.99) in SAVINGS, consisting of:**<br>$0.35 (-0.16 to 0.82) in direct cost savings<br>$8.76 (6.52 to 11.36) in productivity gains

Table H. Incremental cost-effectiveness ratios (ICERs) for the modelled elimination scenarios over 2018-2050 for three economic perspectives.
Costs and DALYs are discounted at 3.5% per annum, with costs presented in 2018 US$. Perspective A includes direct costs only (costs for testing, treatment, and healthcare management). Perspective B includes direct costs (perspective A) plus productivity costs. Perspective C includes partially integrated direct costs and productivity costs. The values represent the median and 95% uncertainty intervals across 1,151 model fits.

	Costs (US$ billions)		DALYs (millions)		ICER	Probability	
**Scenarios until 2050**	**Total**	**Incremental**	**Total**	**Incremental DALYs averted**	**Cost/DALY averted**	**Cost-effective†**	**Cost-saving**
**Perspective A**							
**SQ. Status Quo**	$10.22 (9.16 to 11.33)	--	52.30 (40.17 to 68.36)	--	--	--	--
**EL. Elimination**	$11.28 (10.47 to 12.00)	$1.06 (0.49 to 1.56)	25.76 (20.59 to 31.98)	26.45 (19.31 to 36.70)	$40	100%	0%
**Perspective B**							
**SQ. Status Quo**	$29.87 (24.95 to 35.58)	--	52.30 (40.17 to 68.36)	--	--	--	--
**EL. Elimination**	$22.21 (19.64 to 25.10)	-$7.68 (-10.58 to -5.13)	25.76 (20.59 to 31.98)	26.45 (19.31 to 36.70)	-$284	100%	100%
**Perspective C**							
**SQ. Status Quo**	$28.98 (24.09 to 34.69)	--	52.30 (40.17 to 68.36)	--	--	--	--
**EL. Elimination**	$19.90 (17.36 to 22.81)	-$9.10 (-11.99 to -6.54)	25.76 (20.59 to 31.98)	26.45 (19.31 to 36.70)	-$337	100%	100%

†Compared to estimated empirical health opportunity cost-based willingness-to-pay (WTP) threshold of US$148–198 per DALY averted in 2018 for Pakistan.[28] For all three economic perspectives, the elimination scenario is cost-effective compared to the both the lower and upper limits of the WTP thresholds.

Table I. Details of univariate sensitivity analyses scenarios investigated.

Sensitivity Analysis Scenario	Description
**X1.** No integration of initial screening	Assume no integration of initial screening as in Perspective B, compared to 1/3 integration at baseline.
**X2.** Vary integration of initial screening	Assume integration of initial screening is lower (1/6) or higher (2/3), compared to 1/3 integration at baseline.
**X3.** No healthcare costs pre-cirrhosis	Assume no healthcare costs for pre-cirrhosis disease states compared to including these costs at baseline.
**X4.** Vary cost of productive life year lost	Assume the cost of a productive life lost per year is 20% lower or higher than baseline. Further sensitivity analyses assume that the cost of a productive life lost per year is equal to median income (US$603.91 in 2018) instead of GDP per capita ($1,443.63 in 2018) at baseline. The median income for Pakistan in 2018 was derived by using the reported median income from routinely collected data by Gallup Analytics (reported as US$480 in 2013[29]), which for Pakistan involves annual face-to-face interviews of approximately 1,000 persons conducted in Urdu[29], adjusted to 2018 US$ using the average annual real GDP growth in Pakistan over 2013-2018 (4.7% per annum[30]).
**X5.** Include reduction in absenteeism pre-cirrhosis after SVR	Assume that the reduction in absenteeism following SVR is the same for pre-cirrhosis disease states as for post-cirrhosis disease states (relative reduction of 44% [Range: 30.8-57.2%]), compared to baseline where the reduction in absenteeism following SVR only affects post-cirrhosis disease states.
**X6.** Vary DAA medication cost	Assume that the cost of DAA medication is halved ($9) or doubled ($36) compared to baseline ($18 for 12-weeks).
**X7.** No employment for ESLD	Assume that individuals in end-stage liver disease (ESLD) states, namely DC and HCC, have no employment, compared to baseline in which employment is included for ESLD.
**X8.** No disutility prior to DC	Assume that there is no disutility prior to DC, compared to baseline in which disutility is applied to all disease states.
**X9.** Employment for PWID halved	Assume that the paid employment rate for PWID is half of the male general population, compared to baseline in which they are the same.
**X10.** Vary discounting rate	Assume no (0%) or double (7%) the baseline annual discounting rate (3.5%) applied to costs and outcomes.
**X11.** Achieve HCV elimination target by 2025	Assume that the WHO elimination target of reducing HCV incidence by 80% were to be achieved sooner, by 2025 compared to 2030 (baseline), which could occur, for example, through faster intervention scale-up. This sensitivity analysis scenario assumes that initial one-time screening of the entire population occurs over 3 years (2018-2020 inclusive) instead of over 5 years (2018-2022 inclusive) as in the baseline elimination scenario (EL), as well as 100% referral to treatment for diagnosed individuals (instead of 90% at baseline). Re-testing is the same as in the baseline elimination scenario.

Table J. Univariate sensitivity analyses for the elimination scenario from economic perspective C.
Net economic benefit at 2030 is the negative of the total sum of direct and indirect costs over 2018-2030, with positive values indicating a net monetary gain (bolded entries) and negative values indicating a net monetary loss (see Fig 2b for net economic benefit over time at baseline). The baseline model projections using economic perspective C is shaded. Costs and DALYs are discounted at a rate of 3.5% per annum, with costs presented in 2018 US$. The values represent the median and 95% uncertainty intervals (UI) across 1,151 model fits.

**Scenario**	**Total direct costs 2018-2030 compared to SQ (US$ billions)**	**Total indirect costs 2018-2030 compared to SQ (US$ billions)**	**Net economic benefit at 2030 compared to SQ (US$ billions)**	**Cost per DALY averted at 2030**	**Probability of being cost-saving by 2030**	**Year elimination becomes cost-saving‡**
Baseline (Economic perspective C)	$1.45 (1.32 to 1.60)	-$1.30 (-1.72 to -0.94)	-$0.16 (-0.59 to 0.33)	$29	25.3%	2030.5 (2029.2 to 2032.1)
No integration of initial screening (Economic perspective B)	$2.31 (2.15 to 2.47)	-$1.30 (-1.72 to -0.94)	-$1.01 (-1.45 to -0.52)	$181	0%	2033.0 (2031.3 to 2035.3)
Partial integration is less (integrate 1/6 of initial Ab screening vs. 1/3)	$1.76 (1.62 to 1.91)	-$1.29 (-1.69 to -0.96)	-$0.48 (-0.92 to -0.001)	$85	2.5%	2031.4 (2030.0 to 2033.3)
Partial integration is more (integrate 2/3 of initial Ab screening vs. 1/3)	$0.84 (0.71 to 0.97)	-$1.30 (-1.71 to -0.96)	**$0.47 (0.05 to 0.92)**	-$81	98.7%	2028.6 (2027.6 to 2029.8)
Achieve HCV elimination target for incidence sooner (by 2025 vs. 2030)	$1.40 (1.23 to 1.58)	-$1.79 (-2.34 to -1.30)	**$0.39 (-0.18 to 1.00)**	-$49	88.4%	2029.0 (2027.9 to 2030.6)
No healthcare management costs pre-cirrhosis	$1.94 (1.80 to 2.07)	-$1.30 (-1.71 to -0.95)	-$0.63 (-1.03 to -0.19)	$110	0.5%	2032.1 (2030.5 to 2034.2)
Cost per year of productive life lost is 20% less ($1154.90 vs. $1,443.63)	$1.45 (1.32 to 1.60)	-$1.04 (-1.37 to -0.76)	-$0.41 (-0.76 to -0.03)	$73	1.7%	2031.4 (2030.1 to 2033.1)
Cost per year of productive life lost is 20% more ($1,732.36 vs. $1,443.63)	$1.45 (1.32 to 1.60)	-$1.56 (-2.05 to -1.15)	**$0.11 (-0.37 to 0.65)**	-$18	65.3%	2029.7 (2028.5 to 2031.2)
Cost of productive life lost based on median income ($603.91 vs. $1443.63)	$1.45 (1.32 to 1.60)	-$0.54 (-0.71 to -0.40)	-$0.92 (-1.15 to -0.66)	$166	0%	2034.3 (2032.7 to 2036.6)
Include reduction in absenteeism pre-cirrhosis post-SVR (44% vs. 0%)	$1.45 (1.32 to 1.60)	-$1.45 (-1.92 to -1.05)	-$0.01 (-0.46 to 0.51)	$2	48.5%	2030.0 (2028.7 to 2031.6)
Double DAA medication cost ($36 vs. $18 for 12-weeks)	$1.59 (1.44 to 1.74)	-$1.30 (-1.76 to -0.97)	-$0.29 (-0.71 to 0.22)	$51	12.1%	2030.9 (2029.4 to 2032.5)
Halve DAA medication cost ($9 vs. £18 for 12-weeks)	$1.39 (1.25 to 1.53)	-$1.30 (-1.73 to -0.94)	-$0.10 (-0.51 to 0.38)	$18	34.8%	2030.3 (2029.0 to 2031.9)
No employment if ESLD (0% for DC and HCC vs. fully employed)	$1.45 (1.32 to 1.60)	-$1.20 (-1.56 to -0.88)	-$0.26 (-0.64 to 0.16)	$46	12.7%	2030.8 (2029.6 to 2032.4)
No disutility prior to decompensated cirrhosis	$1.45 (1.32 to 1.60)	-$1.30 (-1.71 to -0.96)	-$0.15 (-0.57 to 0.31)	$29	26.6%	2030.5 (2029.2 to 2032.0)
Employment among PWID is halved (38.6% vs. 77.2%)	$1.45 (1.32 to 1.60)	-$1.30 (-1.71 to -0.95)	-$0.15 (-0.57 to 0.31)	$27	26.3%	2030.5 (2029.2 to 2032.1)
No discounting (0% vs. 3.5%)	1.57 (1.38 to 1.75)	-$1.80 (-2.35 to -1.31)	**$0.23 (-0.33 to 0.87)**	-$29	75.8%	2029.5 (2028.5 to 2030.8)
Double discount rate (7% vs. 3.5%)	1.34 (1.23 to 1.45)	-$0.94 (-1.23 to -0.69)	-$0.40 (-0.71 to -0.06)	$97	1.4%	2031.9 (2030.2 to 2034.1)

‡Year when net economic benefit becomes positive, estimated to nearest one-tenth of a year.

Other Supplementary Materials

Checklist A. Consolidated Health Economic Evaluation Reporting Standards (CHEERS) checklist.[31]

Section/item	Item No.	Recommendation	Reported in section/paragraph
**Title and abstract**			
Title	1	Identify the study as an economic evaluation or use more specific terms such as “cost-effectiveness analysis”, and describe the interventions compared.	Title
Abstract	2	Provide a structured summary of objectives, perspective, setting, methods (including study design and inputs), results (including base case and uncertainty analyses), and conclusions.	Abstract
Introduction			
Background and objectives	3	Provide an explicit statement of the broader context for the study. Present the study question and its relevance for health policy or practice decisions.	Introduction, Paragraphs 1-5
**Methods**			
Target population and subgroups	4	Describe characteristics of the base case population and subgroups analysed, including why they were chosen.	Section **‘HCV transmission model for Pakistan’**; Section **‘Baseline model calibration’**; Fig A
Setting and location	5	State relevant aspects of the system(s) in which the decision(s) need(s) to be made.	Section **‘Model impact and cost-effectiveness analyses’**, Paragraphs 1-4
Study perspective	6	Describe the perspective of the study and relate this to the costs being evaluated.	Section **‘Model impact and cost-effectiveness analyses’**, Paragraphs 5-8
Comparators	7	Describe the interventions or strategies being compared and state why they were chosen.	Section **‘Model impact and cost-effectiveness analyses’**, Paragraphs 1-4
Time horizon	8	State the time horizon(s) over which costs and consequences are being evaluated and say why appropriate.	Section **‘Model impact and cost-effectiveness analyses’**, Paragraphs 1-4
Discount rate	9	Report the choice of discount rate(s) used for costs and outcomes and say why appropriate.	Section **‘Productivity costs due to HCV infection’**, Paragraph 3
Choice of health outcomes	10	Describe what outcomes were used as the measure(s) of benefit in the evaluation and their relevance for the type of analysis performed.	Section **‘Model impact and cost-effectiveness analyses’**, Paragraph 4
Measurement of effectiveness	11a	Single study-based estimates: Describe fully the design features of the single effectiveness study and why the single study was a sufficient source of clinical effectiveness data.	Section **‘HCV transmission model for Pakistan’**; Section **‘Baseline model calibration’**; Section **‘Model impact and cost-effectiveness analyses’**
	11b	Synthesis-based estimates: Describe fully the methods used for identification of included studies and synthesis of clinical effectiveness data.	Not applicable
Measurement and valuation of preference based outcomes	12	If applicable, describe the population and methods used to elicit preferences for outcomes.	Not applicable

Estimating resources and costs	13a	Single study-based economic evaluation: Describe approaches used to estimate resource use associated with the alternative interventions. Describe primary or secondary research methods for valuing each resource item in terms of its unit cost. Describe any adjustments made to approximate to opportunity costs.	Not applicable
	13b	Model-based economic evaluation: Describe approaches and data sources used to estimate resource use associated with model health states. Describe primary or secondary research methods for valuing each resource item in terms of its unit cost. Describe any adjustments made to approximate to opportunity costs.	Section **'Cost and health utility'**; Section **'Productivity costs due to HCV infection'**; Table 1-2; Methods A-B in S1 Text; Fig B
Currency, price date, and conversion	14	Report the dates of the estimated resource quantities and unit costs. Describe methods for adjusting estimated unit costs to the year of reported costs if necessary. Describe methods for converting costs into a common currency base and the exchange rate.	Section **'Productivity costs due to HCV infection'**, Paragraph 3; Table 1
Choice of model	15	Describe and give reasons for the specific type of decision-analytical model used. Providing a figure to show model structure is strongly recommended.	Section **'HCV transmission model for Pakistan'**; Methods A-B in S1 Text; Fig A-B in S1 Text
Assumptions	16	Describe all structural or other assumptions underpinning the decision-analytical model.	Section **'HCV transmission model for Pakistan'**; Section **'Baseline model calibration'**; Methods A-B in S1 Text; Fig A-B in S1 Text
Analytical methods	17	Describe all analytical methods supporting the evaluation. This could include methods for dealing with skewed, missing, or censored data; extrapolation methods; methods for pooling data; approaches to validate or make adjustments (such as half cycle corrections) to a model; and methods for handling population heterogeneity and uncertainty.	Section **'Productivity costs due to HCV infection'**; Section **'Model impact and cost-effectiveness analyses'**; Section **'Sensitivity analyses'**; Methods A-B in S1 Text
**Results**			
Study parameters	18	Report the values, ranges, references, and, if used, probability distributions for all parameters. Report reasons or sources for distributions used to represent uncertainty where appropriate. Providing a table to show the input values is strongly recommended.	Table 1-2; Table A-D in S1 Text
Incremental costs and outcomes	19	For each intervention, report mean values for the main categories of estimated costs and outcomes of interest, as well as mean differences between the comparator groups. If applicable, report incremental cost-effectiveness ratios.	Section **'Impact and cost of status quo HCV treatment scenario'**; Section **'Impact of HCV elimination scenario'**; Section **'Cost of HCV elimination scenario'**; Section **'Cost-effectiveness of HCV elimination scenario'**; Section **'Sensitivity analyses'**; Table 4; Table G-H and Table J in S1 Text
Characterising uncertainty	20a	Single study-based economic evaluation: Describe the effects of sampling uncertainty for the estimated incremental cost and incremental effectiveness	Not applicable

		parameters, together with the impact of methodological assumptions (such as discount rate, study perspective).	
	20b	Model-based economic evaluation: Describe the effects on the results of uncertainty for all input parameters, and uncertainty related to the structure of the model and assumptions.	Section **'Impact and cost of status quo HCV treatment scenario'**; Section **'Impact of HCV elimination scenario'**; Section **'Cost of HCV elimination scenario'**; Section **'Cost-effectiveness of HCV elimination scenario'**; Section **'Sensitivity analyses'**; Table 3; Table E-H and Table J in S1 Text
Characterising heterogeneity	21	If applicable, report differences in costs, outcomes, or cost-effectiveness that can be explained by variations between subgroups of patients with different baseline characteristics or other observed variability in effects that are not reducible by more information.	Not applicable
**Discussion**			
Study findings, limitations, generalisability, and current knowledge	22	Summarise key study findings and describe how they support the conclusions reached. Discuss limitations and the generalisability of the findings and how the findings fit with current knowledge.	Discussion, Paragraphs 1-5; Section **'Strengths and limitations'**; Section **'Comparison with other studies'**; Section **Conclusions**
**Other**			
Source of funding	23	Describe how the study was funded and the role of the funder in the identification, design, conduct, and reporting of the analysis. Describe other non-monetary sources of support.	Funding statement
Conflicts of interest	24	Describe any potential for conflict of interest of study contributors in accordance with journal policy. In the absence of a journal policy, we recommend authors comply with International Committee of Medical Journal Editors recommendations.	Competing Interests statement

Supplementary References

1. Lim, A. G. *et al.* Effects and cost of different strategies to eliminate hepatitis C virus transmission in Pakistan: a modelling analysis. *The Lancet Global Health* **8,** e440–e450 (2020).
2. Grossman, M. On the Concept of Health Capital and the Demand for Health. *Journal of Political Economy* **80,** 223–255 (1972).
3. Younossi, Z. *et al.* Impact of eradicating hepatitis C virus on the work productivity of chronic hepatitis C (CH-C) patients: an economic model from five European countries. *J Viral Hepat* **23,** 217–226 (2016).
4. DiBonaventura, M. D. *et al.* The impact of hepatitis C on labor force participation, absenteeism, presenteeism and non-work activities. *J Med Econ* **14,** 253–261 (2011).
5. World Health Organization. Disease burden and mortality estimates; cause-specific mortality, 2000–2016. Available at: http://www.who.int/healthinfo/global_burden_disease/estimates/en/. (Accessed: 27 October 2020)
6. Central Intelligence Agency. The CIA World Factbook. Available at: https://www.cia.gov/library/publications/the-world-factbook/. (Accessed: 30 June 2016)
7. Finance Division, Government of Pakistan. Pakistan Economic Survey 2014-15. (2015). Available at: http://www.finance.gov.pk. (Accessed: 30 June 2016)
8. United Nations, Department of Economic and Social Affairs, Population Division. United Nations World Population Prospects: The 2015 Revision. (2015). Available at: https://esa.un.org/unpd/wpp/. (Accessed: 30 June 2016)
9. United Nations Office on Drugs and Crime. Drug Use in Pakistan 2013. (2013). Available at: http://www.unodc.org. (Accessed: 30 June 2016)
10. Mathers, B. M. *et al.* Mortality among people who inject drugs: a systematic review and meta-analysis. *Bull. World Health Organ.* **91,** 102–123 (2013).
11. Qureshi, H., Bile, K. M., Jooma, R., Alam, S. E. & Afridi, H. U. R. Prevalence of hepatitis B and C viral infections in Pakistan: findings of a national survey appealing for effective prevention and control measures. *East. Mediterr. Health J.* **16 Suppl,** S15–23 (2010).
12. Micallef, J. M., Kaldor, J. M. & Dore, G. J. Spontaneous viral clearance following acute hepatitis C infection: a systematic review of longitudinal studies. *J Viral Hepat* **13,** 34–41 (2006).
13. Nelson, P. K. *et al.* Global epidemiology of hepatitis B and hepatitis C in people who inject drugs: results of systematic reviews. *Lancet* **378,** 571–583 (2011).
14. van der Meer, A. J. *et al.* Association between sustained virological response and all-cause mortality among patients with chronic hepatitis C and advanced hepatic fibrosis. *JAMA* **308,** 2584–2593 (2012).
15. Morgan, R. L. *et al.* Eradication of hepatitis C virus infection and the development of hepatocellular carcinoma: a meta-analysis of observational studies. *Ann. Intern. Med.* **158,** 329–337 (2013).
16. Kanwal, F., Kramer, J. R., Ilyas, J., Duan, Z. & El-Serag, H. B. HCV genotype 3 is associated with an increased risk of cirrhosis and hepatocellular cancer in a national sample of U.S. Veterans with HCV. *Hepatology* **60,** 98–105 (2014).
17. Thein, H.-H., Yi, Q., Dore, G. J. & Krahn, M. D. Estimation of stage-specific fibrosis progression rates in chronic hepatitis C virus infection: a meta-analysis and meta-regression. *Hepatology* **48,** 418–431 (2008).
18. Grieve, R. *et al.* Cost effectiveness of interferon alpha or peginterferon alpha with ribavirin for histologically mild chronic hepatitis C. *Gut* **55,** 1332–1338 (2006).
19. Shepherd, J., Jones, J. & Hartwell, D. Interferon alfa (pegylated and non-pegylated) and ribavirin for the treatment of mild chronic hepatitis C: a systematic review and economic evaluation. *Health Technology Assessment* (2007).
20. Wright, M. *et al.* Measurement and determinants of the natural history of liver fibrosis in hepatitis C virus infection: a cross sectional and longitudinal study. *Gut* **52,** 574–579 (2003).
21. United States Census Bureau. International Data Base. Available at: http://www.census.gov/population/international/data/idb/. (Accessed: 30 June 2016)
22. European Association for the Study of the Liver. Electronic address: easloffice@easloffice.eu. EASL Recommendations on Treatment of Hepatitis C 2016. *J. Hepatol.* (2016). doi:10.1016/j.jhep.2016.09.001

23. World Health Organization. GUIDELINES FOR THE CARE AND TREATMENT OF PERSONS DIAGNOSED WITH CHRONIC HEPATITIS C VIRUS INFECTION. 1–108 (2019).
24. Amir, M., Rahman, A. S., Jamal, Q. & Siddiqui, M. A. End treatment response and sustained viral response in hepatitis C virus genotype 3 among Pakistani population. *Ann Saudi Med* **33,** 555–558 (2013).
25. Qureshi, S., Batool, U., Iqbal, M., Burki, U. F. & Khan, N. U. Pre-treatment predictors of response for assessing outcomes to standard treatment in infection with HCV genotype 3. *J Coll Physicians Surg Pak* **21,** 64–68 (2011).
26. Umar, M. & Bilal, M. Hepatitis C, a mega menace: a Pakistani Perspective. *J Pioneer Med Sci* (2012).
27. Khaliq, S. & Raza, S. M. Current Status of Direct Acting Antiviral Agents against Hepatitis C Virus Infection in Pakistan. *Medicina (Kaunas)* **54,** (2018).
28. Ochalek, J., Lomas, J. & Claxton, K. Estimating health opportunity costs in low-income and middle-income countries: a novel approach and evidence from cross-country data. *BMJ Glob Health* **3,** e000964 (2018).
29. Galllup Analytics. Country Dataset Details 2005-2020. 1–134 (2021).
30. Finance Division, Government of Pakistan. Pakistan Economic Survey 2019-20. 1–516 (2020).
31. Husereau, D. *et al.* Consolidated Health Economic Evaluation Reporting Standards (CHEERS) statement. *Value Health* **16,** e1–5 (2013).
